# Transcriptome Characteristics and Six Alternative Expressed Genes Positively Correlated with the Phase Transition of Annual Cambial Activities in Chinese Fir (*Cunninghamia lanceolata* (Lamb.) Hook)

**DOI:** 10.1371/journal.pone.0071562

**Published:** 2013-08-12

**Authors:** Zhanjun Wang, Jinhui Chen, Weidong Liu, Zhanshou Luo, Pengkai Wang, Yanjuan Zhang, Renhua Zheng, Jisen Shi

**Affiliations:** 1 Key Laboratory of Forest Genetics and Biotechnology, Ministry of Education of China, Nanjing Forestry University, Nanjing, China; 2 Fujian Academies of Forestry, Southern Mountain Timber Forest Cultivation Lab, the Ministry of Forestry, Fuzhou, China; Karlsruhe Institute of Technology, Germany

## Abstract

**Background:**

The molecular mechanisms that govern cambial activity in angiosperms are well established, but little is known about these molecular mechanisms in gymnosperms. Chinese fir (*Cunninghamia lanceolata* (Lamb.) Hook), a diploid (2*n*  = 2*x*  = 22) gymnosperm, is one of the most important industrial and commercial timber species in China. Here, we performed transcriptome sequencing to identify the repertoire of genes expressed in cambium tissue of Chinese fir.

**Methodology/Principal Findings:**

Based on previous studies, the four stage-specific cambial tissues of Chinese fir were defined using transmission electron microscopy (TEM). In total, 20 million sequencing reads (3.6 Gb) were obtained using Illumina sequencing from Chinese fir cambium tissue collected at active growth stage, with a mean length of 131 bp and a N50 of 90 bp. SOAPdenovo software was used to assemble 62,895 unigenes. These unigenes were further functionally annotated by comparing their sequences to public protein databases. Expression analysis revealed that the altered expression of six homologous genes (*ClWOX1*, *ClWOX4*, *ClCLV1*-*like*, *ClCLV*-*like*, *ClCLE12*, and *ClPIN1*-*like*) correlated positively with changes in cambial activities; moreover, these six genes might be directly involved in cambial function in Chinese fir. Further, the full-length cDNAs and DNAs for *ClWOX1* and *ClWOX4* were cloned and analyzed.

**Conclusions:**

In this study, a large number of tissue/stage-specific unigene sequences were generated from the active growth stage of Chinese fir cambium. Transcriptome sequencing of Chinese fir not only provides extensive genetic resources for understanding the molecular mechanisms underlying cambial activities in Chinese fir, but also is expected to be an important foundation for future genetic studies of Chinese fir. This study indicates that *ClWOX1* and *ClWOX4* could be possible reverse genetic target genes for revealing the molecular mechanisms of cambial activities in Chinese fir.

## Introduction

Wood formation involves the cambium cell activities of division and differentiation, including cell expansion, cell wall thickening, lignification, and programmed cell death [1]–[3]. Cambium cells can maintain themselves periclinally, and they give rise to xylem tissue (wood) centripetally and phloem tissue (bast) centrifugally [4]–[6]. Therefore, an understanding of the regulation of cambial activities could facilitate the improvement of wood yield and quality. However, cambial activity is a complex process, which is regulated by both genetic and environmental signals [7]–[9]. While previous studies have focused mainly on anatomical, biochemical, and cytological aspects [4], [10]–[14], the molecular mechanisms of cambial activities are not well understood. Fortunately, with the completion of genome sequences for model plants such as *Arabidopsis* [15] and *Populus* [16], many molecular mechanisms and genes involved in cambial activity, such as *TDIF/CLE41/CLE44-TDR/PXY-WOX4* [17]–[22], *Class III HD-Zip/KANADI* [23]–[26], have been identified in angiosperms [27]. Unfortunately, little is known about the molecular mechanisms and genes in gymnosperms. There has been a rapid development of genomic and molecular tools, especially next-generation sequencing (NGS) technologies, such as the Illumina Genome Analyzer, Roche 454 GS FLX Titanium, and ABI SOLiD [28]–[30], which have been widely applied to transcriptome sequencing in cambium and other vascular tissues, such as *Eucalyptus* [31], *Liriodendron* [32], *Acacia* [33], and so on. In addition, the altered expression of many regulatory genes correlates strongly with changes in cambial activity [34], and indeed many such genes are highly expressed when the cambium is active. Hence, the molecular mechanisms of cambial activity in gymnosperms can be revealed using NGS in the active growth stage of the cambium zone.

Nonetheless, as a result of the structural characteristics and number of features of cambium cells [35], [36], cambium tissue is difficult to identify accurately and sample successfully [37], [38]. For instance, actively growing cambial cells are characterized by large central vacuoles, thin tangential walls, and more cell layers. By contrast, dormant cambial cells are characterized by numerous subdivided vacuoles, thick tangential walls, and fewer cell layers [35], [36]. Laser microdissection (LMD), a technique for collecting cell- or tissue-specific material [39], has been successfully applied in sampling of cambium in *Populus* [40]–[42] and *Picea glauca* [43]. However, the amount of RNA collected using this method often does not meet the requirements of NGS; moreover, LMD has not been wildly used with woody plants [43]. Thus, currently cambium tissues are currently collected primarily using the following methods: (i) scraping the inner surface of fresh bark [44], [45]; (ii) scraping the debarked surface of immature xylem of living trees [46], [47]; (iii) simultaneously scraping cambium tissue using both methods (i) and (ii) [48], [49]. With the return of cambial activity in early springtime, the bark of Chinese fir is easily removed from the tree stem, with the separation occurring at the cambial layer [46]. The cambium tissues can then be scraped from the exposed surface of the xylem [46].

Chinese fir (*Cunninghamia lanceolata* (Lamb.) Hook) is an evergreen coniferous tree that primarily distributed in southern China and northern Vietnam [50]. It is one of the most important coniferous species [51]–[53], with high yield, excellent wood quality, versatile uses, and pest resistant [53], [54]. However, as of February 2012, only 220 nucleotides, 445 expressed sequence tags (ESTs) [52], and 85 proteins from Chinese fir had been deposited in the US National Center for Biotechnology Information (NCBI) GenBank database. In conclusion, the complex genetic background and limited genomic information available in this species are obstacles to understanding the molecular mechanisms on cambial activity.

In this study, we used TEM to define four stage-specific cambium tissues and used transcriptome sequencing to identify genes that are activated during cambial growth. Based on a bioinformatics analysis of assembled transcriptome data, housekeeping gene selection for qRT-PCR and expression analysis of 17 orthologous genes were performed in four stage-specific cambium tissues. Two genes that showed positive correlations with changes in cambial activity were further cloned and analyzed.

## Results

### Anatomical Observations of the Cambium

**Figure 1:**
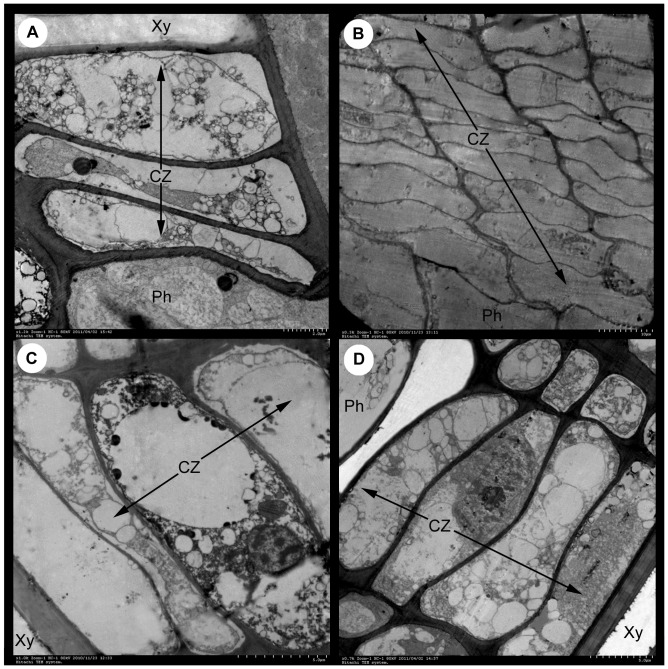
Analysis of cambium ultrastructure. The photographs display the transverse section of second vascular tissue that contains phloem (Ph) and xylem (Xy). CZ, Cambial Zone. The following stages are shown: (A) Reactivation (February 28, stage 1), (B) active growth (May 26, stage 2), (C) transition to dormancy (October 12, stage 3), and (D) dormancy (January 17, stage 4). Bars  = 2.0 µm (A), 10.0 µm (B), and 5.0 µm (C, D).

The four stages of cambial activity were defined by comparing the results of TEM analysis between our study and Peng’s [55] as follows: reactivation (February 28, S1), active growth (May 26, S2), transition to dormancy (October 12, S3), and dormancy (January 17, S4) (**Figures** **1A–D**). The structure of the cambium in the four stages can be described as follows: during S1, the cambial zone consists of three layers of cells containing dense cytoplasm and many small vacuoles (**Figure 1A**); during S2, the cambial zone has nine layers of cells containing a large central vacuole, an organelle-rich cytoplasm, and a thin cell wall (**Figure 1B**); during S3, the cambial zone has three layers of cells containing dense cytoplasm and a large central vacuole and some small vacuoles (**Figure 1C**); during S4, the cambial zone has three to four layers of cells containing dense cytoplasm (**Figure 1D**). These results provided a cytological foundation for further study of the molecular aspects underlying cambial activity.

### Assembly of Sequences

To identify key genes involved in cambial development in Chinese fir, transcriptome sequencing was performed using Illumina sequencing technology. Since the expression of related genes of cambial development is strongly correlated with the activity of cambium cells (which are vigorous during activated stage of cambial development), activated stage Chinese fir cambium tissue was used to construct the transcriptome sequencing library (**Figure S1**). The library was sequenced with Illumina HiSeq 2000. A total of 20 million sequencing reads (3.6 Gb) were generated. After filtering out low quality reads, all reads were assembled by SOAPdenovo [56]. The longest assembled sequences containing no Ns were labeled contigs (**Figure S2**). Mapping reads back to contigs and combining paired-end information linked contigs into scaffolds. Scaffold length was estimated according to average segment length of each pair of reads. Unknown bases were filled with Ns. After filling gaps in scaffolds using paired-end reads, we obtained sequences called unigenes. Using information analysis of mapping reads back to contigs and paired-ends, we obtained 637,458 contigs, with a mean length of 131 bp and N50 of 90 bp, the length distributions of these contigs are shown in Table 1; 96.46% of the contigs displayed a length from 75 to 400 bp. Contig joining and gap filling were used to assemble 118,391 scaffolds with an average length of 336 bp and total length of 11.84 Mb (**Table 1**). Finally, 62,895 unigenes were generated from the cambium transcriptome, with an average length of 505 bp, an N50 of 613 bp, and a total length of 31.75 Mb (**Table 1**). There were 37,499 unigenes (59.62%) with lengths varying from 200 to 400 bp, 19,188 unigenes (30.51%) with lengths ranging from 401 to 1000 bp, and 6256 unigenes (9.87%) with lengths of more than 1000 bp (**Table 1**).

**Table 1:** Length distribution of assembled contigs and unigenes.

Nucleotide length (bp)	Contigs	Unigenes
75**–**100	500,245	0
100**–**200	77,620	409
201**–**300	26,115	25,464
301**–**400	10,939	11,626
401**–**500	6300	6673
501**–**600	4069	4329
601**–**700	2824	3048
701**–**800	1996	2154
801**–**900	1465	1666
901**–**1000	1154	1318
1001**–**1200	1614	1863
1201**–**1400	1032	1232
1401**–**1600	671	866
1601**–**1800	465	629
1801**–**2000	332	466
>2000	617	1152
Total	637,458	62,895
N50 (bp)	90	613
Mean length (bp)	131	505
Total nucleotides length (bp)	83,701,046	31,748,167

### Quality Analysis of Assembled Unigenes

**Figure 2:**
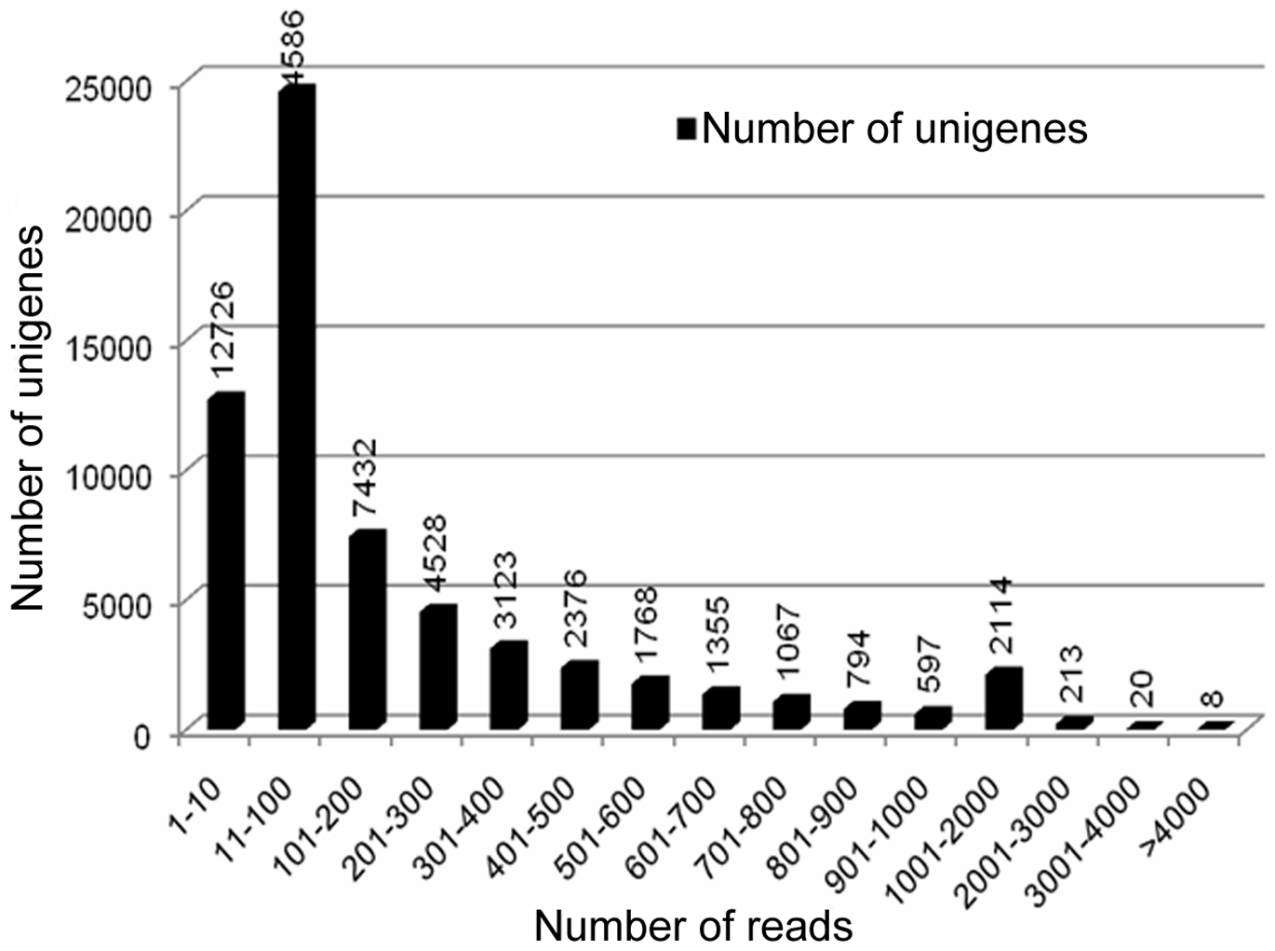
Distribution of uniquely mapped reads of assembled unigenes. The horizontal axis indicates the number of reads. The vertical axis indicates the number of assembled unigenes.

To assess the quality and coverage of assembled unigenes, we analyzed the sequencing depth range. The sequencing depth ranged from 0.19 to 2517 folds, in which 79.71% of the unigenes were more than 10 reads, 40.50% of the unigenes were more than 100 reads, and 20.29% of the unigenes varied from 1 to 10 reads (**Figure 2**).

**Figure 3:**
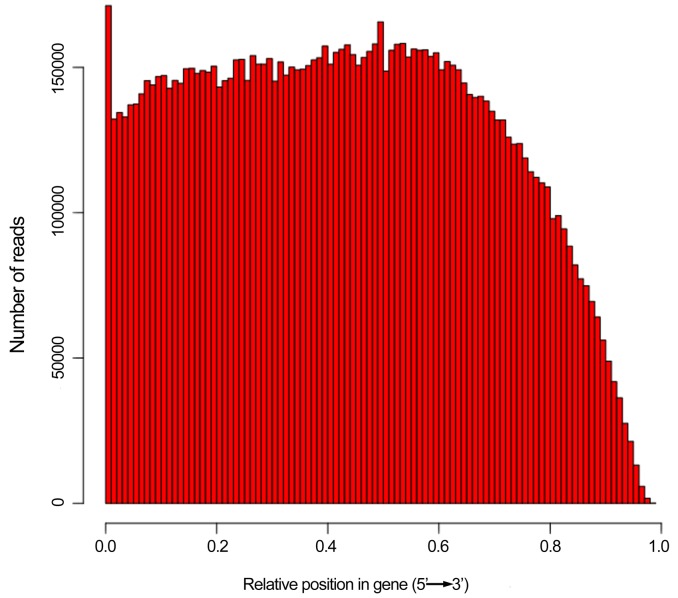
Random distribution of HiSeq 2000 sequencing reads in the assembled unigenes. The horizontal axis indicates relative position in gene (5'→3'). The vertical axis indicates the number of assembled reads.

**Figure 4:**
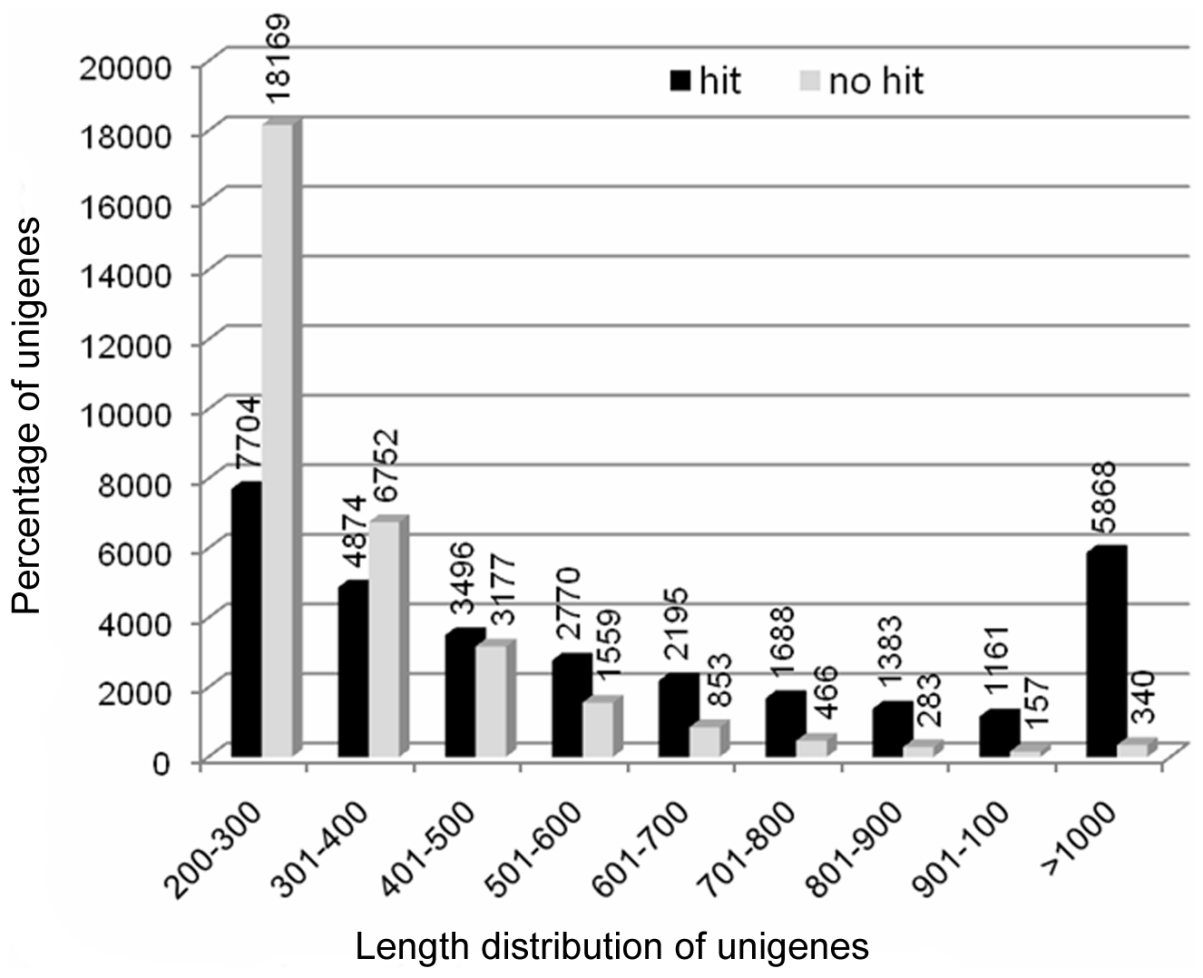
Comparison of length distribution between hit and no-hit unigenes. The horizontal axis indicates length distribution of hit and no-hit assembled unigenes. The vertical axis indicates the percentage of hit and no-hit assembled unigenes.

To further analyze sequencing bias, random distribution of reads was detected in unigenes. Interestingly, comparison of the location of reads among the 5′ ends and other positions of all unigenes showed that the reads seldom appeared near the 3' ends of all unigenes (**Figure 3**). In addition, the length of unigenes was compared between hit and no-hit in protein databases by BLAST matches. Consequently, 70.63% of unigenes over 500 bp in length had BLAST matches, 45.74% of unigenes between 300 bp and 500 bp had BLAST matches, whereas only 29.78% of unigenes shorter than 300 bp had BLAST matches (**Figure 4**). These results were similar to previous reports of transcriptome research on *Ipomoea batatas* [57] and *Camellia sinensis* [58]. To detect the sequence similarity in gene level between Chinese fir and *Pinus taeda*, TBLASTX alignment was performed on Chinese fir transcriptomes against a draft genome sequence of *Pinus taeda*. 21,281 unigenes (∼33.84% of all the 62,895 unigenes) of Chinese fir had significant matches in the *Pinus taeda* genome sequence (**Table S1**). Taken together, these results demonstrated that the quality of the Chinese fir unigenes was indeed high.

### Functional Annotation

**Figure 5:**
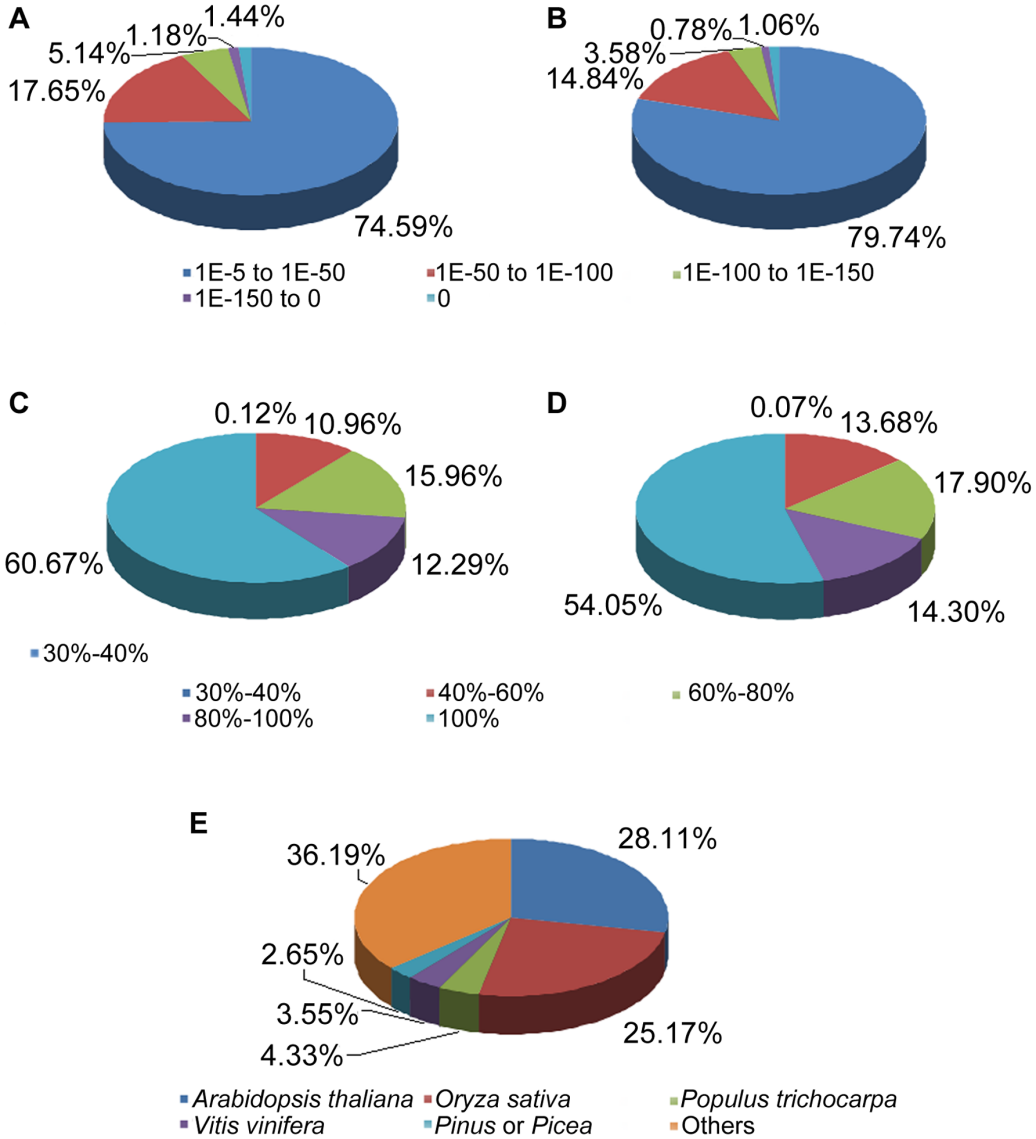
Assigned distribution of unigenes. E value (A, B), similarity (C, D), and species (E) distributions using results from the Nr (A, C, E) and Swiss-Prot (B, D, E) databases. All unigenes were blasted with a cut-off E value of 1E^−5^.

To predict putative functions of the assembled unigenes, we compared all unigenes with public protein databases, namely NCBI non-redundant protein (Nr), Swiss-Prot protein, Clusters of Orthologous Groups (COG), Gene Ontology (GO), and the Kyoto Encyclopedia of Genes and Genomes (KEGG) (**Figure S2**) [59]. Among the 62,895 unigenes, 27,629 and 18,157 were identified in Nr and Swiss-Prot (**Table 2**), respectively. The *E* value, similarity, and species distributions of the annotated results were compared with Nr and Swiss-Prot. Nr and Swiss-Prot results respectively showed that 74.59% (20,609) and 79.74% (14,478) of unigenes had high homology (*E* value range 10^–5^ to 10^–50^), and 60.67% (16,761) and 54.05% (9815) showed 100% similarity (**Figures 5A–D**). The species distribution analysis showed that the Chinese fir unigenes were more closely related to sequences of *Arabidopsis* (8754, 28.11%) and *Oryza* (7838, 25.17%) than to *Populus* (1349, 4.33%), *Vitis* (1104, 3.55%), and *Pinus* or *Picea* (824, 2.65%) (**Figure 5E**). These results underscore the paucity of reference genomes and incompletely defined genetic backgrounds for gymnosperms [60].

**Table 2:** Summary of the percentage of unigenes annotated for Chinese fir.

Database	Number of unigenes	Percent of unigenes annotated
Nr	27,629	43.93
Swiss-Prot	18,157	28.87
COG	15,662	24.90
GO	24,791	39.42
KEGG	14,402	22.90

**Figure 6:**
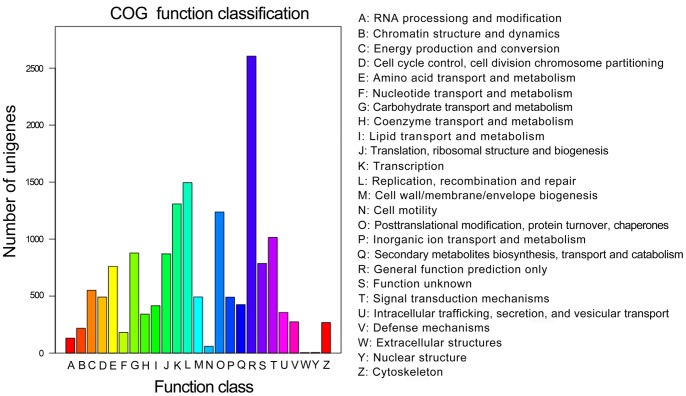
COG functional classification of the transcriptome. A total of 15,662 unigenes were classified into 25 COG categories.

All unigenes were compared with the COG database, and 15,662 unigenes of the Chinese fir transcriptome had COG classifications (**Figure 6** **,** **Tables 2** **, S2**). Among the 25 COG categories, the cluster for ‘general function prediction only’ (2605, 16.63%) represented the largest group, followed by ‘replication, recombination and repair’ (1495, 9.55%), ‘transcription’ (1308, 8.35%), ‘posttranslational modification, protein turnover, chaperones’ (1238, 7.90%), and ‘signal transduction mechanisms’ (1015, 6.48%). There were only a few annotated unigenes for the categories ‘extracellular structures’ (4, 0.03%) and ‘nuclear structure’ (5, 0.03%) (**Table S2**).

**Figure 7:**
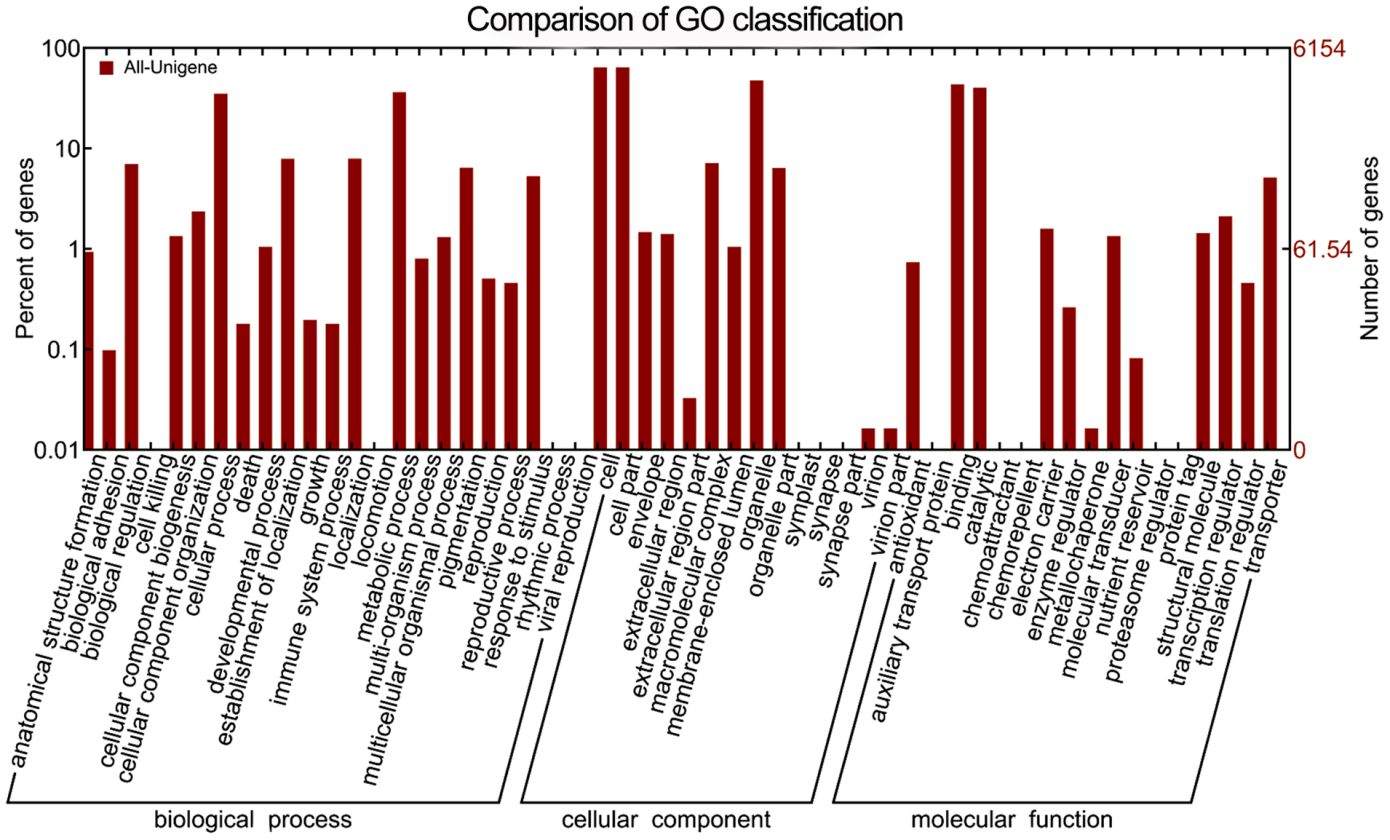
GO classification of the transcriptome. GO classification of the Chinese fir transcriptome. The horizontal axis indicates the three main categories that include molecular function, cellular component, and biological process, and certain categories that are self evident. The horizontal axis indicates the three main categories that include molecular function, cellular component, and biological process, and certain categories that are self evident. The right vertical axis indicates the number of assembled unigenes in each category, and the left vertical axis indicates the percentage of a certain type of subcategory in that main category.

Blast2GO (Nr annotation) was applied to assign GO categories [61], and WEGO software [62] showed that 24,791 unigenes of the Chinese fir transcriptome had GO classifications (**Table 2**), including ‘cellular component’ (11,819) was a more dominant GO functional classification of the annotated unigenes than the ‘biological process’ (7051) and ‘molecular function’ (5921). The cellular component category was mostly composed of ‘cell’ (3923, 63.75%), ‘cell part’ (3922, 63.73%), and ‘organelle’ (2902, 47.16%). The ‘biological process’ consisted mostly of the ‘metabolic process’ (2220, 36.07%) and ‘cellular process’ (2147, 34.89%), and ‘molecular function’ was comprised mostly of ‘binding’ (2659, 43.21%) and ‘catalytic’ (2457, 39.93%) (**Figure 7** **, Table S3**). These results were similar to those published for non-model plant transcriptomes [57], [58].

A total of 14,402 annotated sequences of the cambium transcriptome were assigned to 125 KEGG reference canonical pathways (**Tables 2** **, S4**). The pathways most represented were ‘secondary metabolites’ (5277), ‘amino acid metabolism’ (1693), ‘plant-pathogen interaction’ (1197), ‘carbohydrate metabolism’ (1183), and ‘transcription’ (1093) (**Table 3**). These results partially explained why a large number of secondary metabolites exist in Chinese fir.

**Table 3:** Mapping of assembled unigenes of the Chinese fir transcriptome to KEGG pathways.

KEGG pathways	KEGG sub-pathways	Number of unigenes
Metabolism	Carbohydrate metabolism	1183
	Lipid metabolism	824
	Amino acid metabolism	1693
	Nucleotide metabolism	605
	Glycan biosynthesis and degradation	191
	Metabolism of cofactors and vitamins	232
	Secondary metabolites	5277
Genetic information processing	Replication and repair	660
	Transcription	1093
	Folding, sorting and degradation	206
	Translation	210
Plant-pathogen interaction		1197

### Coding Sequence Prediction and Expression Analysis

To further infer unigene function for Chinese fir, we identified unigene coding sequences and searched protein databases using BLASTX (*E* value <10^−5^) in the following order: Nr, Swiss-Prot, KEGG, and COG. If unigenes were matched in one database, they were not further analyzed against another database. These BLAST results were applied to collect coding DNA sequence information from unigenes and then translate them into peptide sequences. Unigenes with no matches in BLASTX (using ESTScan) [63] were employed to predict coding DNA sequences and then translate them into peptide sequences. Finally, 30,818 and 4037 unigenes had their coding DNA sequences identified by BLASTX and ESTScan, respectively. Expression of all unigenes was denoted by reads per kilobase of exon model per million mapped reads (RPKM) values [64]. Table 4 lists the top 10 most frequently expressed unigenes in the cambial transcriptome. The average RPKM value for all unigenes was 30.16.

**Table 4:** The top 10 most frequently expressed unigenes in the Chinese fir transcriptome.

Rank	Unigene ID	RPKM value	Function annotation
1	Unigene 4299	1005.0917	Ribosomal protein S7
2	Unigene 19270	731.2727	Mtn21-like protein
3	Unigene 26045	687.1027	4-coumarate:CoA ligase
4	Unigene 5166	595.8491	Unknown
5	Unigene 11581	583.4117	Plasma intrinsic protein 2,1
6	Unigene 33274	583.1480	Malate dehydrogenase
7	Unigene 23196	576.9831	Lactoylglutathione lyase
8	Unigene 13199	570.8247	Actin
9	Unigene 18523	566.9721	Ubiquitin
10	Unigene 15174	561.1256	UPF0727 protein WS02710_H03

### *eIF-3* was a Stable Housekeeping Gene for qRT-PCR in Four Stage-specific Cambiums

To obtain reliable results from qRT-PCR analysis, the ideal reference gene (internal control gene) should be selected in samples regardless of different tissues, developmental stages, and/or sample treatment [65]–[67], and the combination of geNorm [68], NormFinder [69], and BestKeeper [67], [70], [71] software that had been applied to identify reference genes from the transcriptome data [71], [72]. We selected nine housekeeping genes from the transcriptome data on Chinese fir cambium, and identified their expression by RT-PCR before qRT-PCR analysis (**Table S5**). The analysis of expressional stability in the nine housekeeping genes was carried out by qRT-PCR. geNorm, NormFinder, and BestKeeper were used to analyze the results. geNorm evaluated housekeeping gene results from most stable to least stable: *ClUBQ<CleIF-4A*<*CleIF-3*<*ClActin*<*ClEF-1α*<*ClGAPDH* <*Cl40S*<*Clβ-TU*<*Clα-TU* (**Figure S3, Table S6**). NormFinder analyzed housekeeping genes from most stable to least stable: *ClActin<CleIF-4A*<*ClUBQ* <*CleIF-3*<*ClEF-1α*< *ClGAPDH*<*Cl40S*<*Clβ-TU*<*Clα-TU* (**Figure S4, Table S6**). BestKeeper described the housekeeping genes from most stable to least stable: *ClGAPDH*<*CleIF-3*<*ClEF-1α*<*ClUBQ* <*CleIF-4A*<*ClActin* <*Cl40S*<*Clα-TU*<*Clβ-TU* (**Figure S5, Table S6**). Considering that the difference found with BestKeeper is larger than the other results, we chose the *CleIF-3* as the most appropriate reference genes for qRT-PCR analysis of cambial development in Chinese fir.

### The Altered Expression of Six Genes was Correlated Positively with Changes in Cambial Activity

To identify genes in the Chinese fir transcriptome database that regulate cambial activity, 17 highly expressed genes (RPKM value >30.16) (**Tables 5** **, S7**) that are known homologs of model-plant regulatory genes were selected as candidates to perform expression analysis of stages S1–S4 in cambium tissues. In angiosperms, these regulatory genes play important roles in controlling cambium initiation and development [20]–[22], [34], [73], [74], vascular development and differentiation [6], [20], [40], [75]–[77], and plant stem cell fate [20]–[22], [40], [77]. Therefore, it is interesting to examine the functional conservation of these regulatory genes between angiosperms and gymnosperms. The 17 candidate genes could be categorized as follows: (i) *WOX* and *CLV* genes: *ClWOX1*, *ClWOX4*, *ClWOX8*, *ClWOX9*, *ClCLV1*, *ClCLV1-like*, *ClCLV2*, *CLV-like*, and *ClCLE12*; (ii) *ClassIII HD-Zip* genes: *ClREV*, *ClPHB1*, and *ClATHB15*; (iii) hormone-related genes: *ClPIN1-like*, *ClAUX*, and *ClARR7*; and (iv) *ClSHR* and *ClSCR*.

**Table 5:** The 17 homologous genes used for qRT-PCR.

Gene	Unigene ID	RPKM value	Putative annotation	ID	*E* value
ClWOX1	Unigene 56359	90.27	WUSCHEL-related homeobox 1	gi|61217290|	2.00E-06
ClWOX4	Unigene 30112	144.58	WUSCHEL-related homeobox 4	gi|30693997|	4.00E-29
ClWOX8	Unigene 58521	145.76	WUSCHEL-related homeobox 8	gi|75286325|	2.00E-53
ClWOX9	Unigene 3729	38.05	WUSCHEL-related homeobox 9	gi|61217281|	7.00E-13
ClCLV1	Unigene 20219	34.14	CLAVATA1	gi|290796119|	3.00E-91
ClCLV1-like	Unigene 26065	35.74	CLAVATA1-like	gi|290882856|	1.00E-22
ClCLV2	Unigene 60215	39.28	CLAVATA2	gi|6049567|	1.00E-92
ClCLV-like	Unigene 57886	32.16	CLAVATA-like	gi|104642235|	5.00E-56
ClCLE12	Unigene 59778	36.69	CLE12 (CLAVATA3/ESR-RELATED 12)	gi|18409123|	1.00E-06
ClREV	Unigene 22939	57.94	REVOLUTA	gi|75203823|	1.00E-14
ClPHB1	Unigene 56464	133.88	PHB1	gi|71370257|	1.00E-98
ClATHB15	Unigene 38884	111.27	ATHB-15	gi|75216693|	5.00E-45
ClSHR	Unigene 60570	30.89	SHR	gi|75213595|	2.00E-90
ClSCR	Unigene 48261	37.52	SCARECROW	gi|112012486|	2.00E-54
ClPIN1-like	Unigene 38615	123.70	PIN1-like auxin transport protein	gi|25956262|	1.00E-39
ClAUX	Unigene 13481	412.34	Auxin influx transport protein	gi|151564283|	4.00E-42
ClARR7	Unigene 60762	65.09	Two-component response regulator ARR7	gi|51316125|	2.00E-39

**Figure 8:**
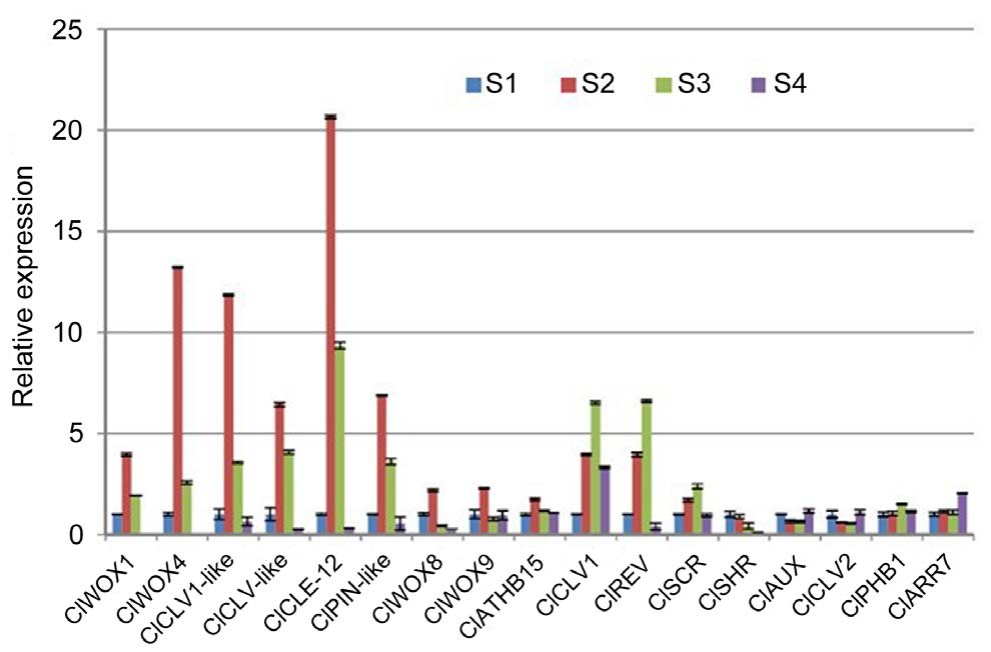
Expression analysis of 17 homologous genes in cambium tissues during the four growth stages, S1–S4, using qRT-PCR. Error bars represent the standard error of three biological replicates.

Comparison of the results of the gene expression analysis (**Figure 8**) and the histological analysis (**Figure 1**) revealed that the observed altered expression of the 17 candidates could be classified into three groups. The first group (*ClWOX1*, *ClWOX4*, *ClCLV1-like*, *ClCLV-like*, *ClCLE12*, *ClPIN1-like*, *ClWOX8*, *ClATHB15*, and *ClWOX9*) was expressed most highly during S2. Six genes (*ClWOX1*, *ClWOX4*, *ClCLV1-like*, *ClCLV-like*, *ClCLE12*, and *ClPIN1-like*) were upregulated during S1 and S2 and downregulated during S3 and S4. Interestingly, *ClWOX4* expression increased 415-fold between S2 and S4. The second group (*ClCLV1*, *ClREV*, and *ClSCR*) was expressed most highly during S3. In the third group, the expression of *ClSHR* gradually decreased from S1 to S4, and that of *ClAUX* correlated negatively with cambial activity. These results revealed that the altered expression of six candidate genes, namely *ClWOX1*, *ClWOX4*, *ClCLV1-like*, *ClCLV-like*, *ClCLE12*, and *ClPIN1-like*, correlated positively with changes in cambial activity; moreover, these six genes might be directly involved in cambial function in Chinese fir.

### Molecular Characterization of *ClWOX1* and *ClWOX4*

The altered expression of six genes (*ClWOX1*, *ClWOX4*, *ClCLV1-like*, *ClCLV-like*, *ClCLE12*, and *ClPIN1-like*) was correlated positively with changes in cambial activity. *WOX* genes perform similar regulation functions in the plant embryo development of both gymnosperms and angiosperms [78]–[80], and it has been revealed that *WOX4* can regulate the cambial activity in angiosperms [21]. In addition, *WOX1* has also been identified with regulating lateral organ development in angiosperms [81], [82]. *ClWOX1* and *ClWOX4* were selected to clone from among the six genes mentioned in the preceding paragraph. The gene structure of *ClWOX1* and *ClWOX4* are displayed in Figure S6. These results will facilitate phylogenetic analyses and structural comparison of the *WOX* gene family in gymnosperms and angiosperms. As shown in Figure S7, WUS homodomain and WUS box sequences are similar to the results of the previous study showing that the multiple alignment of amino acid sequences predicted WOX proteins [82], [83] (**Figure S7**). Based on the full amino acid sequences of 108 *WOX* homologs, a phylogenetic tree could be resolved into the following three subgroups: *WUS*, *WOX9*, *WOX13* lineage [82]–[84] (**Figure S8**). *ClWOX1* and *ClWOX4* were assigned to the *WUS* lineage. *ClWOX1* was closely related to *PSWUS* (*Pinus*) and *AtWOX6* (*Arabidopsis*). *ClWOX4* was more closely related to *PsitWOX4* (*Pinus sitchensis*).

## Discussion

For conifers with very large genomes (18 to 35 Gb) [85], studies of wood formation and vascular tissue activity have focused on transcript profile investigations in conifer trees to date [86]. Moreover, recent development of NGS technologies provides revolutionary tools for detecting transcriptome especially with the advent of RNA-sequencing (RNA-seq) [87]. For example, Illumina, 454, and SOLiD have been widely applied on NSG platforms [28]–[30], [87]–[89]. However, each sequencing technology has its own advantage and disadvantage [87]–[89]. For example, SOLiD increases the accuracy of sequencing results against double error correction, but with the longest run times [88], [89]. 454 has the advantage of longer reads, fast run times, good choice for *de novo* assembly, but existing higher reagent costs and error rates in homopolymeric tracts [88], [89]. The Illumina technology has high coverage, cost-effectiveness, and high-throughput, but inherent shorter read-lengths, less feasible for *de novo* assembly [88], [89]. Specially, Illumina HiSeq 2000 has been shown that it is currently the most widely used NGS platform [89]. In the present study, we used Illumina HiSeq 2000 to perform transcriptome sequencing for activated stage cambium of Chinese fir. 20 million sequencing reads (3.6 Gb) were generated.

The quality of transcriptome data is a decisive factor for various downstream analyses [90]. However, the quality of the data may be affected by several factors, including contaminant of adapter/primer sequences, poor quality reads, and assembly errors [90]. In this study, a total of 62,895 unigenes were assembled using SOAPdenovo software, with an average length of 505 bp and a N50 of 613 bp. For non-model plants, owing to absence of homology unigenes, the average length of unigenes after assembly from Illumina transcriptome sequencing is usually less than 500 bp, such as is the case with *Camellia sinensis* (mean = 355 bp) [58], *Dendrocalamus latiflorus* (mean = 461 bp) [91], *Hevea brasiliensis* (mean = 485 bp) [92]. Surprisingly, Chinese fir unigenes have an average length of 505 bp. Moreover, comparison analysis of Chinese fir and *Pinus taeda* in gene sequence similarity also suggested that the quality of our results was credible. However, each assembly strategy has an effect on the accuracy of transcriptome data [87], [93]. To further improve the accuracy of assembled Chinese fir transcriptome sequences, comparative study of different assembly software tools for NGS technologies are worthy of special attention in future analysis.

QRT-PCR is a common means of confirming the expression pattern and the quality of transcriptome data [94]. The ideal reference gene is essential for the accurate measurement in qRT-PCR [67]. Our transcriptome database presumably provides a great potential source of candidate reference genes. Through comprehensive analysis of geNorm, NormFinder, and BestKeeper, *CleIF-3* was identified as the most appropriate reference gene for qRT-PCR analysis during cambial development in Chinese fir. *CleIF-3* not only supplies a housekeeping gene for gene expression analysis in cambium, but also considers as the potential reference gene for gene expression analysis in samples regardless of different tissues, developmental stages, and/or sample treatment. In 2012, Hossain *et al.* indicated that *eEF-1Bβ1* (locus Atlg30230) is involved in plant growth and cell elongation, and plays an important role in plant development and cell wall biosynthesis in *Arabidopsis* [95]. In the future, we should examine the functional conservation of housekeeping genes between Chinese fir and *Arabidopsis*.

*WUSCHEL* (*WUS*)-related homeobox (*WOX*) genes, with 15 members in *Arabidopsis*, play an important role in controlling plant stem cell fate and organogenesis [20]–[22], [80], [81]. In *Arabidopsis*, *WOX4* can promote the proliferation of procambial/cambial stem cells [20]–[22]. In petunia (*Petunia*×*hybrida*), an ortholog of *Arabidopsis WOX1* (*MAEWEST*, *MAW*) and *CHORIPETALA SUZANNE* (*CHSU*) regulates petal fusion and leaf development, and *WOX1* and *PRESSED FLOWER* (*PRS*) redundantly show a similar function in *Arabidopsis* [81]. Although the functions of *ClWOX1* and *ClWOX4* need to be more accurately determined to verify whether they indeed regulate cambial activity in Chinese fir, our results suggest that these two genes may regulate cambial activity. These results also demonstrate that the quality of the transcriptome data is sufficient for gene cloning, and provide insight for further research on stage-specific cambial activity. In addition, previous studies showed that *WOX2*, *WOX8* and *WOX9* have similar function in embryonic growth both in gymnosperms (*Picea abies*) and angiosperms (*Arabidopsis*) [78], [79]. Thus, we have initiated transgenic analysis of the *ClWOX1* and *ClWOX4* in *Arabidopsis*. The functional verification of *ClWOX1* and *ClWOX4* genes, which correlated positively with changes in cambial activity, can help to further understand the mechanisms of cambial activity in Chinese fir. Furthermore, cloning and characterization of the remaining four genes (*ClCLV1-like*, *ClCLV-like*, *ClCLE12*, and *ClPIN1-like*) that are positively correlated with changes in cambial activity will also enhance the understanding into the molecular mechanisms of cambial activity of Chinese fir. This study should provide insight into the development and utilization of transcriptome data.

Our conclusions for this study are as follows: (i) the transcriptome data and expression information for Chinese fir cambium reported herein provide a basis for further studies of the molecular mechanisms that govern cambial activity of gymnosperms. This information will help to increase timber yield and quality of Chinese fir. (ii) This transcriptome database also provides a valuable genetic resource for gene discovery, functional genomics, and comparative genomics of Chinese fir. Moreover, these sequences provided reference sequences for comparative transcriptome analysis in related species. (iii) In addition, the Chinese fir transcriptome database can be applied to the synthesis of gene chips or analysis of digital gene expression (DGE) that can help to study gene expression networks and further confirm the complexity of cambial activity mechanisms [96]. (iv) Furthermore, application of the transcriptome database to the development of molecular markers such as simple sequence repeats (SSRs) [57], [92], [97] will provide helpful information for improvement of wood yield and quality in the third-generation breeding cycle of Chinese fir. In conclusion, our results provide new insights into the mechanisms of Chinese fir cambial activities, and can also be useful for gene discovery, expression profiling, and functional genomics studies of non-model plants in future.

## Materials and Methods

### Ethics Statement

All necessary permits were obtained for the described field studies. The permission for each location was approved by Yang Kou Forest Farm (Fujian, China).

### Plant Materials

The plant materials used for this study were 7-year-old specimens of Chinese fir (average height, 8 m; average diameter at chest height, 17 cm) that belonged to the ramets of a superior clone No. 6421. The specimens were cultivated at a third-generation breeding orchard, which was located on the 4 forest class 2 class 2-class (26°50′ N, 117°54′ E) of the Daoping work area of the Yang Kou Forest Farm, Fujian Province, China. Rootstocks of Chinese fir were colonized in 2002. Scions of clone No. 6421 of Chinese fir were grafted in 2003.

Several stems were cut off at approximately 1 m below the top of the main stem of clone No. 6421 of Chinese fir at 08∶00 a.m. on seven different dates: February 28, April 26, May 26, July 21, August 21, October 12, 2010, and January 17, 2011 (**Figure S9A**). To assess cambium anatomy, tissue blocks [∼3 cm (longitudinal)×3 cm (radial)×3 cm (tangential)] taken from the stems were immediately placed in fresh cold fixative [4% (v/v) glutaraldehyde, 2% (w/v) paraformaldehyde, 0.2 M sodium phosphate buffer (pH 7.2)]. Nine replicates were performed for each stage (3 tissue blocks×3 fixatives).

Simultaneously, for transcriptome sequencing and expression analysis, cambium tissues were collected from cut stems as follows: first, the bark and phloem of the stem were peeled off; next, the cambium tissue was gently scraped from the exposed surface of the woody area with a razor blade (**Figure S9B**) [46], [48] until scraping reached the fibrous tissue below the differentiating cells (**Figure S9B**) [49]; finally, to avoid RNA degradation, the cambium tissues were immediately stored in RNA*later*® (Ambion, Austin, TX, USA) preservation solution (**Figure S9C**), transported to the laboratory within 36 h, and stored at –80°C until use. To eliminate contamination of RNA enzymes, all tools and gloves were treated with RNase*Zap*® (Ambion, Austin, TX, USA) solution. To avoid the impact of sample differences, none of the individual ramets were sampled more than twice, and both samples were taken from the tree at a similar height and at a similar diameter at chest height.

### TEM Analysis

After excess wood and bark were removed from the above-mentioned tissue blocks (∼3×3×3 cm), the remaining tissues were further sectioned (∼4×2×1 mm) and then immediately immersed in the same cold fixative, vacuum infiltrated, and fixed at 4°C for an additional 30 h. The tissues were washed four times with cold buffer (20 min each) and then post-fixed in 1% (w/v) OsO_4_ in 0.2 M sodium phosphate buffer (pH 7.2) overnight at 4°C [11]. The tissues were then dehydrated in a 10% incremental graded series of cold acetone (30 min each) and embedded in Spurr’s resin [14]. Samples were cut into ultrathin sections on an LKB-V ultramicrotome. Ultrathin sections were then stained with uranyl acetate and lead citrate [55]. Finally, 27 ultrathin sections (3 tissue blocks×3 fixatives×3 technical replicates) were observed and photographed for each stage with a Hitachi H-7650 (Tokyo, Japan) TEM at 80 kV. High-quality printing and repeatability of images were selected to record in this article.

### RNA Isolation

Activated stage cambium was selected for transcriptional sequencing to identify more genes associated with cambial activity. Total RNA was isolated from activated stage cambium using an RNA purification kit (Norgen Biotek, ON, Canada) according to the manufacturer’s instructions. Total RNA was purified with RNase-free DNase I (TaKaRa Biotech, Dalian, China) to remove genomic DNA. The RNA quality was verified using the Agilent 2100 Bioanalyzer (Agilent Tecnologies, Santa Clara, CA, USA) in terms of concentration (>300 ng/µL), RNA integrity number (RIN >8.0), and the 28S:18S ratio (1.5). Purified total RNA was stored at –80°C until use. Finally, a total of 20 µg of RNA was used for the transcriptome library construction.

### Transcriptome Library Construction

Each sample for transcriptome sequencing was prepared according to manufacturer’s instructions (**Figure S1**). Illumina HiSeq™ 2000 beads with oligo(dT) were used to isolate poly(A) mRNA from total RNA. Fragmentation buffer was added to create short mRNA fragments (200–700 bp), which were used as templates with random hexamer (N6) primers (Illumina) to synthesize the first-strand cDNA. The second-strand cDNA was synthesized using buffer, dNTPs, RNase H, and DNA polymerase I. Short fragments were purified with a QiaQuick PCR extraction kit and resolved with EB buffer for end reparation and addition of poly(A). The short fragments were then connected with sequencing adapters. For PCR amplification, suitable fragments were selected as templates, according to the results of agarose gel electrophoresis. Finally, the transcriptome library was paired-end sequenced through a Illumina HiSeq™ 2000 platform at the Beijing Genomics Institute (BGI)-Shenzhen (Shenzhen, China) following the manufacturer’s instructions. The sequencing data were deposited in NCBI Sequence Read Archive (SRA, http://www.ncbi.nlm.nih.gov/Traces/sra) with accession number SRA092144.

### *De novo* Assembly

First, raw reads were filtered using stringent requirements to obtain high-quality sequences before assembly analysis (**Figure S2**). Empty reads, having only 3'-adaptor and single-base repeat sequences, were eliminated from raw reads. The others low quality reads were signified for ‘N’. SOAPdenovo associated reads and the specific length of overlap (http://soap.genomics.org.cn/soapdenovo.html) [56] to form longer fragments without N. Such assembled sequences were called contigs. Then the reads were mapped back to contigs with paired-end reads. Contigs were detected from the same transcript as well as the distances between these contigs. Furthermore, ‘N’ was used to denote unknown sequences between each two contigs when we connect the contigs by SOAPdenovo [56]. At that point, scaffolds were created. Paired-end reads were used again to carry out gap-filling of scaffolds to obtain sequences with the least amount of ‘Ns’ that could not be extended on either end. These assembled sequences were defined as unigenes. Furthermore, we evaluated the quality of assembled unigenes after summarizing the assembled results, describing gap distribution, sequencing depth range and distribution of unique mapped reads of the assembled unigenes. Finally, the alignment of unigenes was performed by TBLASTX against the draft genome sequence of *Pinus taeda* (genome size is approximately 22.56 Gb, http://dendrome.ucdavis.edu/NealeLab/lpgp/, Version 0.9). Genome screening was conducted by searching for shared segments based on sequence similarity from initial 60%-threshold in a range of 30 amino acids.

### Bioinformatics Analysis

The assembled data were annotated for function using protein similarity analysis [58]. BLAST alignment (E value <10^–5^) was performed between the unigene set and the following protein databases: Nr (http://www.ncbi.nlm.nih.gov), Swiss-Prot (http://www.expasy.ch/sprot), COG (http://www.ncbi.nlm.nih.gov/COG/), GO (http://www.geneontology.org), and KEGG (http://www.genome.ad.jp/kegg/) [59]. The best aligning results were selected to annotate the transcriptome and to determine the sequence of directions of unigenes. When the aligning results are opposite each other among different databases, the sequence direction of unigenes should be determined using the order of the prior database order (Nr, Swiss-Prot, KEGG, and COG) [59]. However, if a unigene is not aligned to any of the former databases, ESTScan software [63] can be introduced to predict its coding regions and decide its sequence direction, which shows high efficiency in low-quality sequences [98]. The annotated unigenes were normalized in RPKM to calculate their relative expression levels [64].

### Housekeeping Gene Selection for qRT-PCR in Four Stage-specific Cambiums

RNA was isolated and purified from four stage-specific cambiums as described in section 4.4. First-strand cDNA was synthesized using the ReverTra Ace qPCR RT kit (Toyobo, Japan). Nine commonly used housekeeping genes (e.g., *Actin*, *EF-1α*, *eIF-3*, *eIF-4A*, *GAPDH*, *α-TU*, *β-TU*, *40S*, and *UBQ*) were chosen from transcriptome data to perform housekeeping gene selection in four stage-specific cambiums. Primers for these genes were designed by Primer Express Software V3.0 (Applied Biosystems, Foster City, CA, USA) with melting temperatures of 60±2°C and 100- to 150-bp PCR amplicon lengths (**Table S5**). QRT-PCR was performed with an ABI 7500 Real-Time PCR system (Applied Biosystems) using Power SYBR® Green PCR Master Mix (Applied Biosystems) according to the manufacturer’s protocol. Each reaction contained 10 µL of 2× SYBR Green PCR buffer, 1 µL of each specific primer (10 µM), and 1 µL of reverse-transcribed cDNA (∼100 ng) in a final volume of 20 µL and was amplified under the protocol’s conditions: 50°C incubation for 2 min, 95°C incubation for 10 min, followed by 40 PCR cycles consisting of 95°C for 15 s and 60°C for 1 min (http://www.ucl.ac.uk/wibr/services/docs/sybr.pdf). Three biological replicates were carried out for each qRT-PCR reaction. To ensure the specificity of PCR amplification, PCR products were verified using determination of a dissociation curve from 65°C to 95°C after final amplication [99]. geNorm [69], NormFinder [68], and BestKeeper software (**Figures S3, S4, S5, Table S6**) [67], [70], [71] were applied to identify stable housekeeping gene from nine commonly used housekeeping genes.

### Expression Analysis of 17 Orthologous Genes in Four Stage-specific Cambiums

Based on a combination of regulatory genes for cambial activity in angiosperms and RPKM analysis of Chinese fir transcriptome data [64], 17 highly expressed orthologous genes at the active growth stage were subjected to expression analysis using qRT-PCR in four stage-specific cambiums. Primer design and qRT-PCR analysis were performed as described in section 4.7 (**Table 5**). The relative expression level of each gene was calculated using the 2^-ΔΔ*C*t^ method [100]. Based on a combination of geNorm, NormFinder, and BestKeeper analysis (**Figures S3, S4, S5, Table S6**), all quantifications of 17 orthologous genes were normalized to the expression level of *eIF-3*, which was the most stable reference gene for qRT-PCR analysis of Chinese fir cambium from stages S1 to S4 (**Table S7**).

### Molecular Cloning and Sequences Analysis of *ClWOX1* and *ClWOX4*

According to the transcriptome data for Chinese fir, the full-length cDNAs for the *ClWOX1* and *ClWOX4* were cloned from cambium samples by rapid amplification of cDNA ends (RACE) (Clontech, CA, USA) with appropriate RACE primers (**Table S8**). Genomic DNA was isolated from the Chinese fir cambium using the CTAB method [99], [101]. The full-length DNAs for *ClWOX1* and *ClWOX4* were cloned by PCR with whole-sequence primers (**Table S8**). The ORFs of putative full-length cDNAs were predicted using the ORF finder program (http://www.ncbi.nlm.nih.gov/gorf/gorf.html). The genomic schematic diagram of ClWOX1 and ClWOX4 were displayed with Gene Structure Display Server (http://gsds.cbi.pku.edu.cn/) [102]. Homology searchers were carried out by BLASTN and BLASTP (http://www.ncbi.nlm.gov/blast/). Multiple sequence alignment of WOX proteins was performed using Clustal X version 2.1 (**Table S9**) [103]. A phylogenetic tree was constructed with the program MEGA5 [104] by the neighbor-joining method [105], and bootstrap values were estimated by distance analysis for 1,000 replicates, and PsitWOX13 was used as an outgroup.

## Supporting Information

Figure S1 **Experiment pipeline of transcriptome sequencing.**
(TIF)

Figure S2 **Pipeline of bioinformatics analysis.**
(TIF)

Figure S3 **geNorm ranking of candidate reference genes and pairwise variation (V) to determine the optimal number of reference genes.** (A) Average expression stability values for the remaining control genes. The horizontal axis indicates the rank of nine reference genes from least stable to most stable (light®right). The vertical axis indicates the average expression stability value. (B) Determination of the optimal number of control genes for accurate normalization. Pairwise variation (Vn/n+1) was analyzed between the normalization factors NFn and NFn+1 by geNorm software. The horizontal axis indicates the pairwise variation (Vn/n+1) of nine reference genes. The vertical axis indicates the expression stability value.
(TIF)

Figure S4 **NormFinder ranking of reference genes.** The horizontal axis indicates nine reference genes. The vertical axis indicates the expression stability value.
(TIF)

Figure S5 **BestKeeper ranking of reference genes.** The horizontal axis indicates nine reference genes. The vertical axis indicates the Pearson’s correlation coefficient (r) value.
(TIF)

Figure S6 **Schematic representation of structural features of** ***ClWOX1*** **and** ***ClWOX4*** **.** UTR, untranslated region.
(TIF)

Figure S7 **Multiple alignment of amino acid sequences predicted from Chinese fir** ***ClWOX1*** **and** ***ClWOX4*** **cDNAs.**
(TIF)

Figure S8 **Phylogenetic analysis of** ***ClWOX1*** **and** ***ClWOX4*** **.** A neighbor-joining was inferred from the predicted protein sequence of *ClWOX1* and *ClWOX4* with the other *WOX* family proteins using MEGA 5.0. See Supplemental Table S8 for the protein names and accession numbers of 108 WOX.
(TIF)

Figure S9 **Sampling of Chinese fir cambial tissue.** (A) A 7-year-old clone, No. 6421, of Chinese fir. (B) Scraping off the cambial tissues. (C) Collecting and preserving cambial tissues. Bars  = 50 cm (A) and 2 cm (B and C).
(TIF)

Table S1 **Summary of genome screening of Chinses fir transcriptome unigenes against the draft genome sequence of** ***pinus taeda*** **.**
**(XLS)**

Table S2 **COG functional annotation mapping of assembled unigenes of the Chinese fir transcriptome.**
(DOC)

Table S3 **GO mapping of assembled unigenes of the Chinese fir transcriptome.**
(DOC)

Table S4 **Mapping of assembled unigenes of the Chinese fir transcriptome to KEGG pathways.**
(DOC)

Table S5 **Candidate genes used to select housekeeping genes.**
(DOC)

Table S6 **Ranking of candidate genes based on their stability value using geNorm and NormFinder, and their Pearson’s correlation coefficient (r) using BestKeeper.**
(DOC)

Table S7 **The qRT-PCR primers for the 17 homologous genes.**
(DOC)

Table S8 **Primers for cloning** ***ClWOX1*** **and** ***ClWOX4*** **.**
(DOC)

Table S9 **Protein names and accession numbers of 108 WOX.**
(DOC)
