# Supplementary figures and images for: Transcriptome Characteristics and Six Alternative Expressed Genes Positively Correlated with the Phase Transition of Annual Cambial Activities in Chinese Fir (Cunninghamia lanceolata (Lamb.) Hook)
Source: PLoS One. 2013 Aug 12;8(8):e71562. doi: 10.1371/journal.pone.0071562 (PMC3741379; doi:10.1371/journal.pone.0071562)

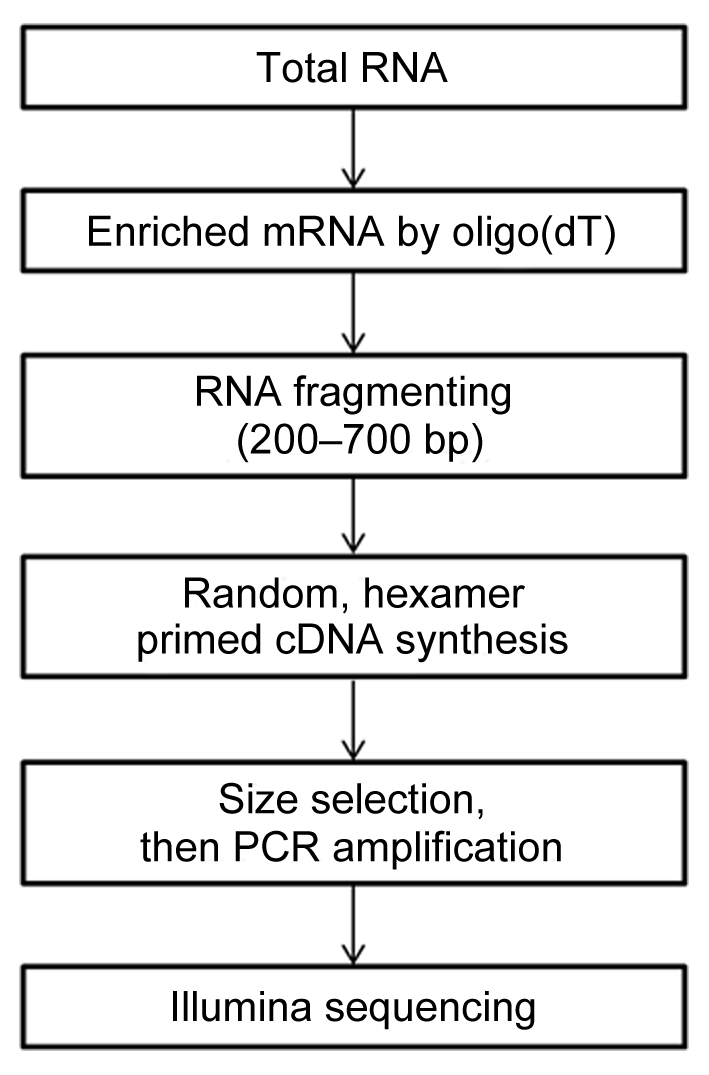

Supplement: Figure S1 — Experiment pipeline of transcriptome sequencing. (TIF) [file pone.0071562.s001.tif]

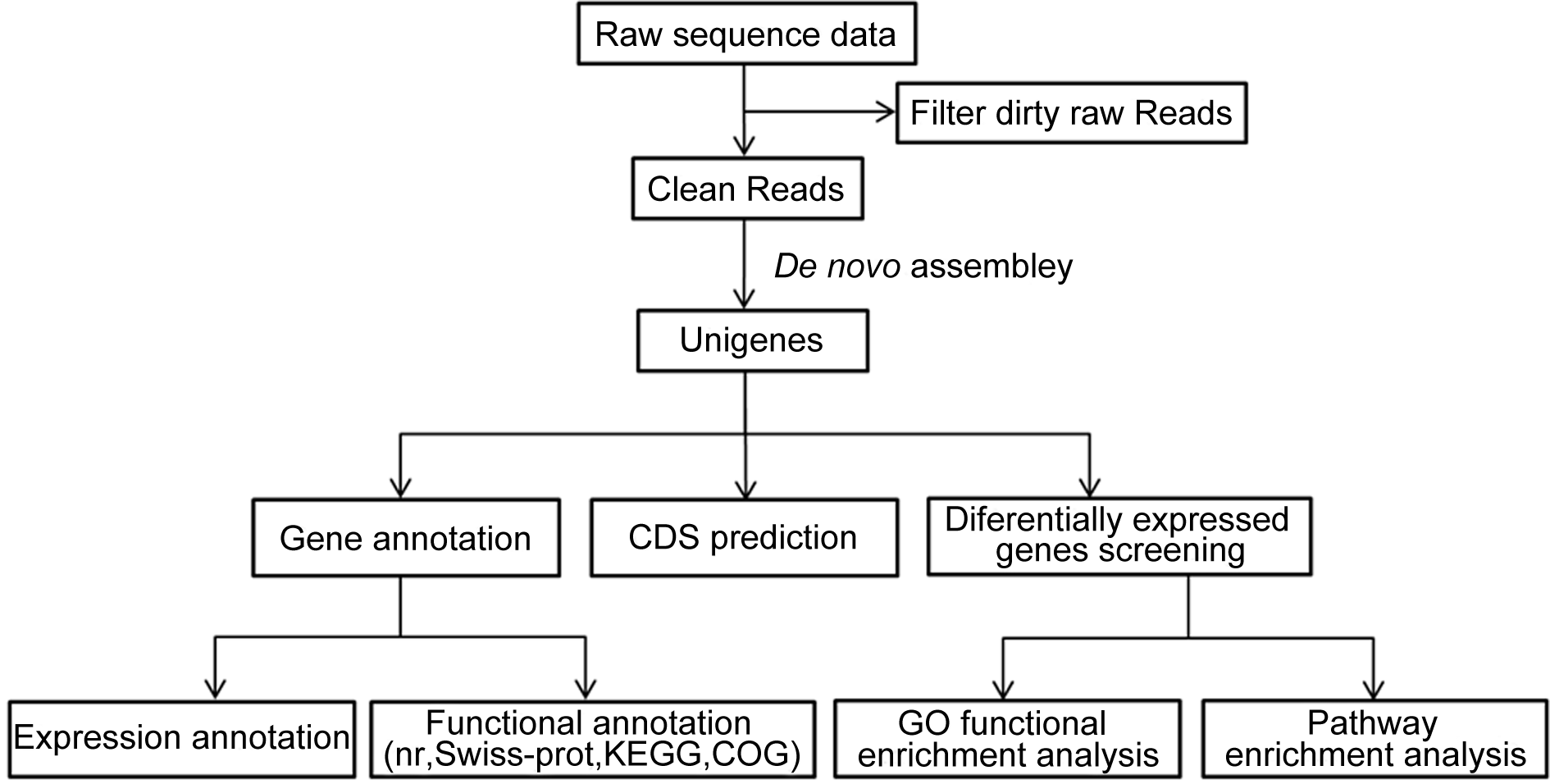

Supplement: Figure S2 — Pipeline of bioinformatics analysis. (TIF) [file pone.0071562.s002.tif]

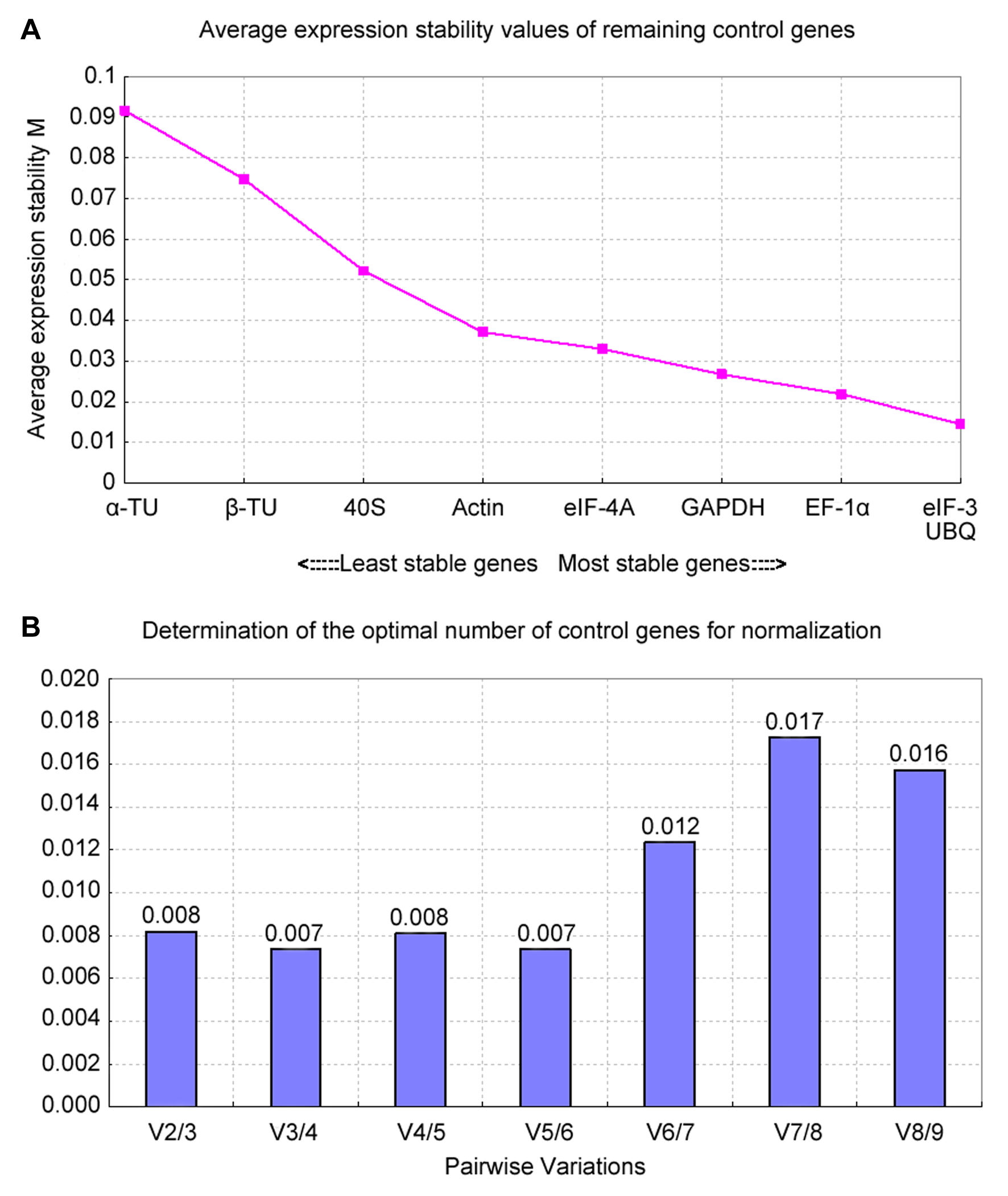

Supplement: Figure S3 — geNorm ranking of candidate reference genes and pairwise variation (V) to determine the optimal number of reference genes. (A) Average expression stability values for the remaining control genes. The horizontal axis indicates the rank of nine reference genes from least stable to most stable (light®right). The vertical axis indicates the average expression stability value. (B) Determination of the optimal number of control genes for accurate normalization. Pairwise variation (Vn/n+1) was analyzed between the normalization factors NFn and NFn+1 by geNorm software. The horizontal axis indicates the pairwise variation (Vn/n+1) of nine reference genes. The vertical axis indicates the expression stability value. (TIF) [file pone.0071562.s003.tif]

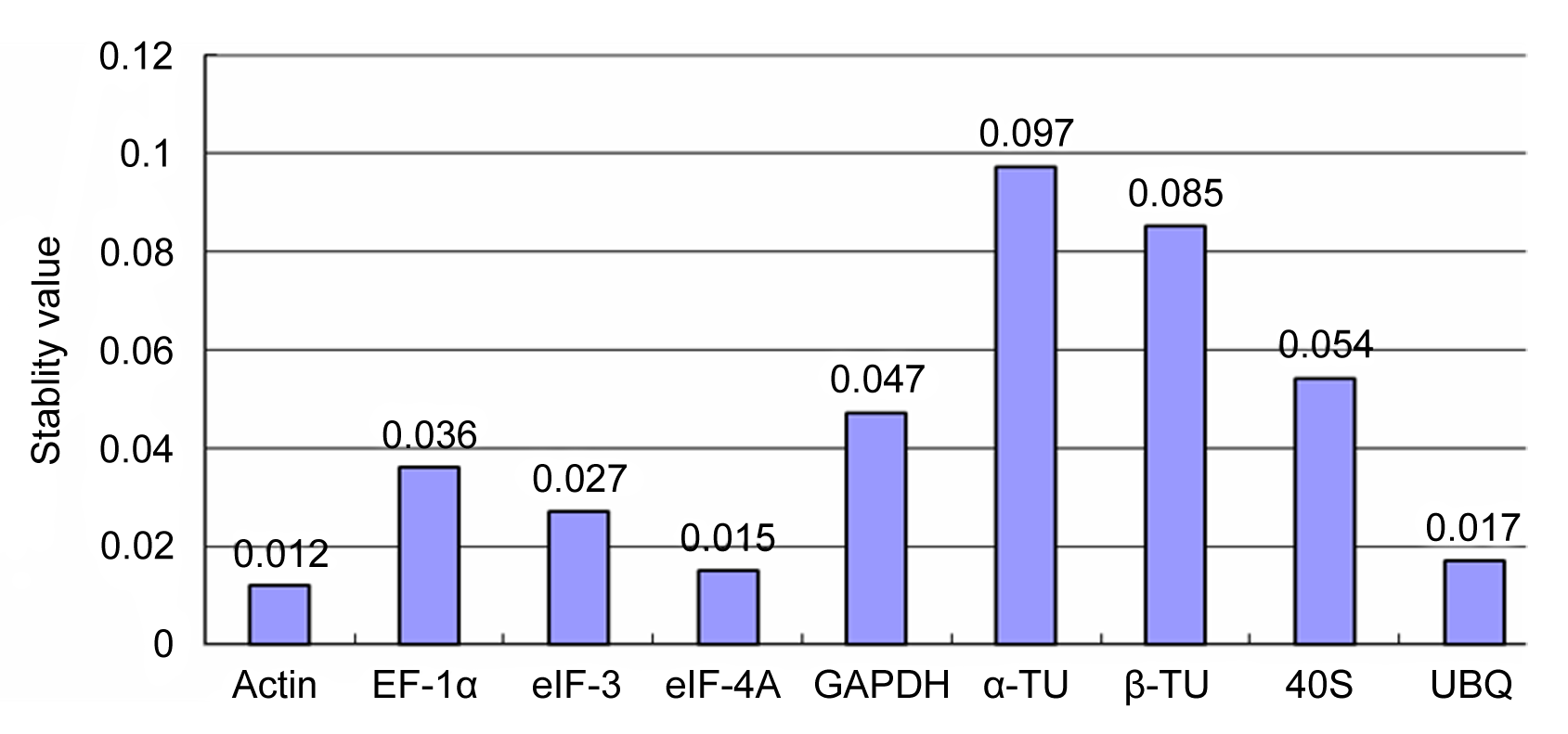

Supplement: Figure S4 — NormFinder ranking of reference genes. The horizontal axis indicates nine reference genes. The vertical axis indicates the expression stability value. (TIF) [file pone.0071562.s004.tif]

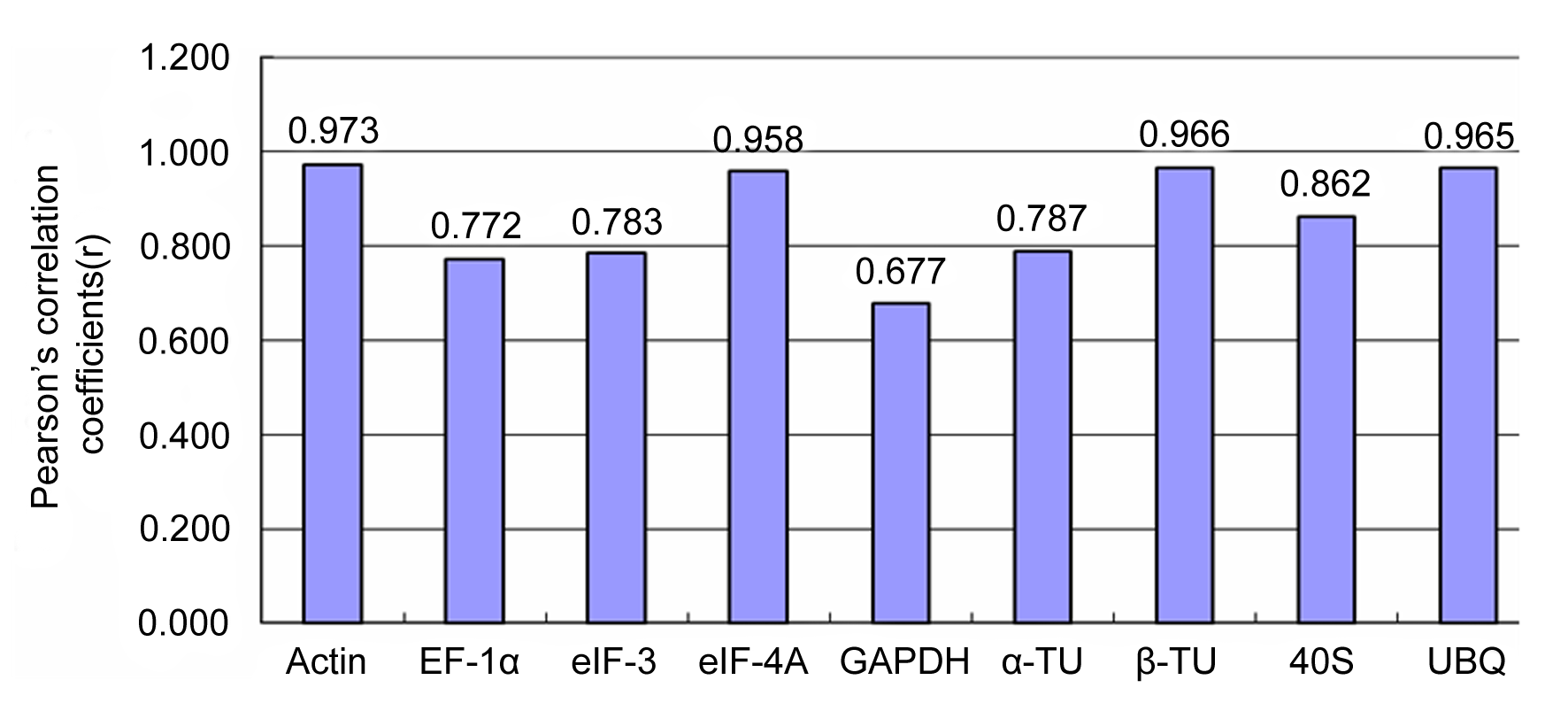

Supplement: Figure S5 — BestKeeper ranking of reference genes. The horizontal axis indicates nine reference genes. The vertical axis indicates the Pearson’s correlation coefficient (r) value. (TIF) [file pone.0071562.s005.tif]

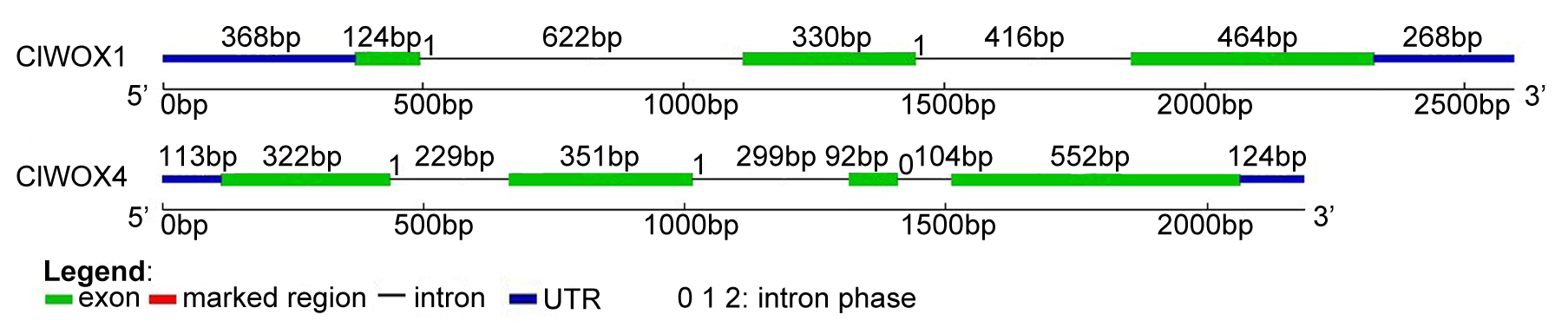

Supplement: Figure S6 — Schematic representation of structural features of ClWOX1 and ClWOX4 . UTR, untranslated region. (TIF) [file pone.0071562.s006.tif]

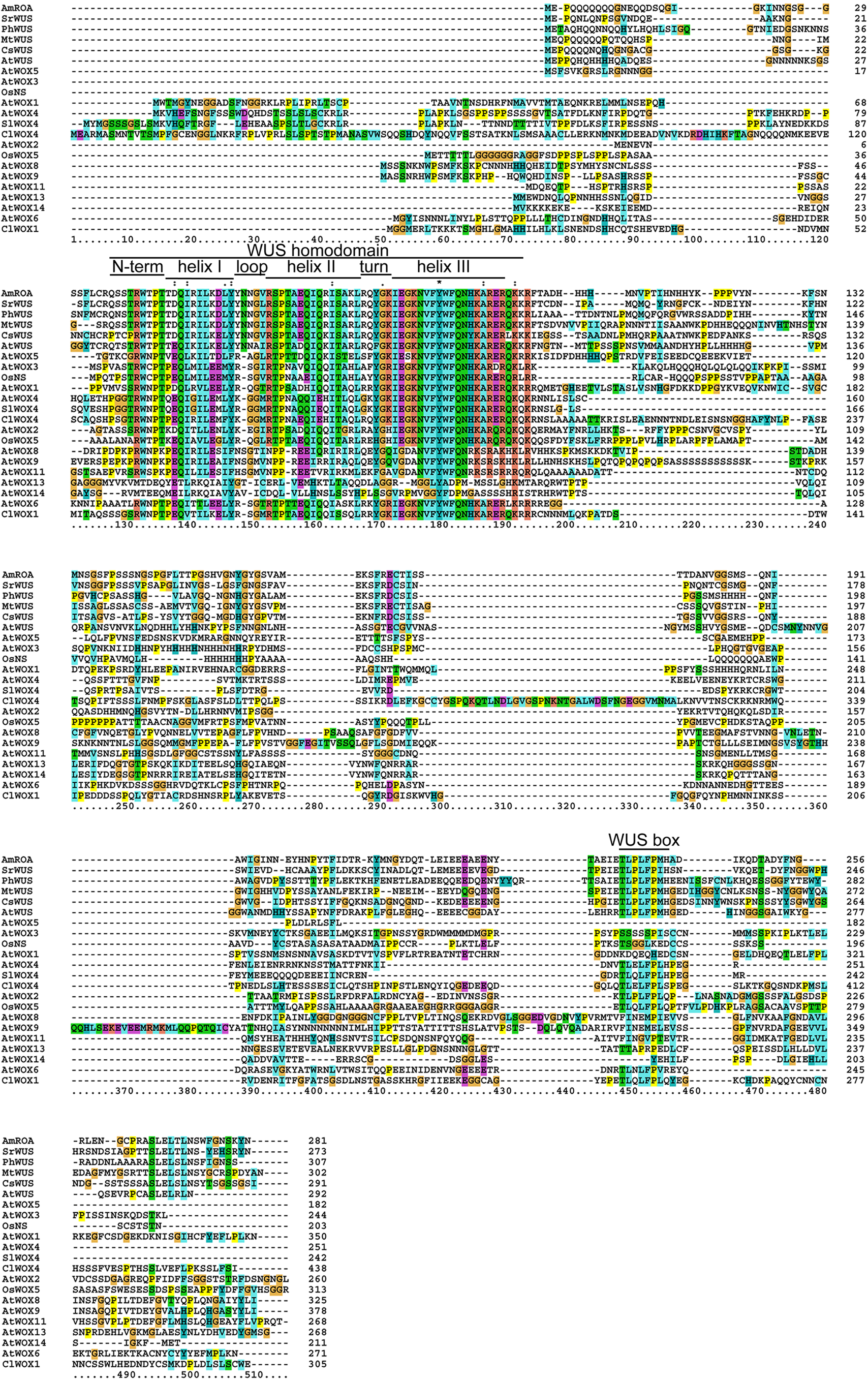

Supplement: Figure S7 — Multiple alignment of amino acid sequences predicted from Chinese fir ClWOX1 and ClWOX4 cDNAs. (TIF) [file pone.0071562.s007.tif]

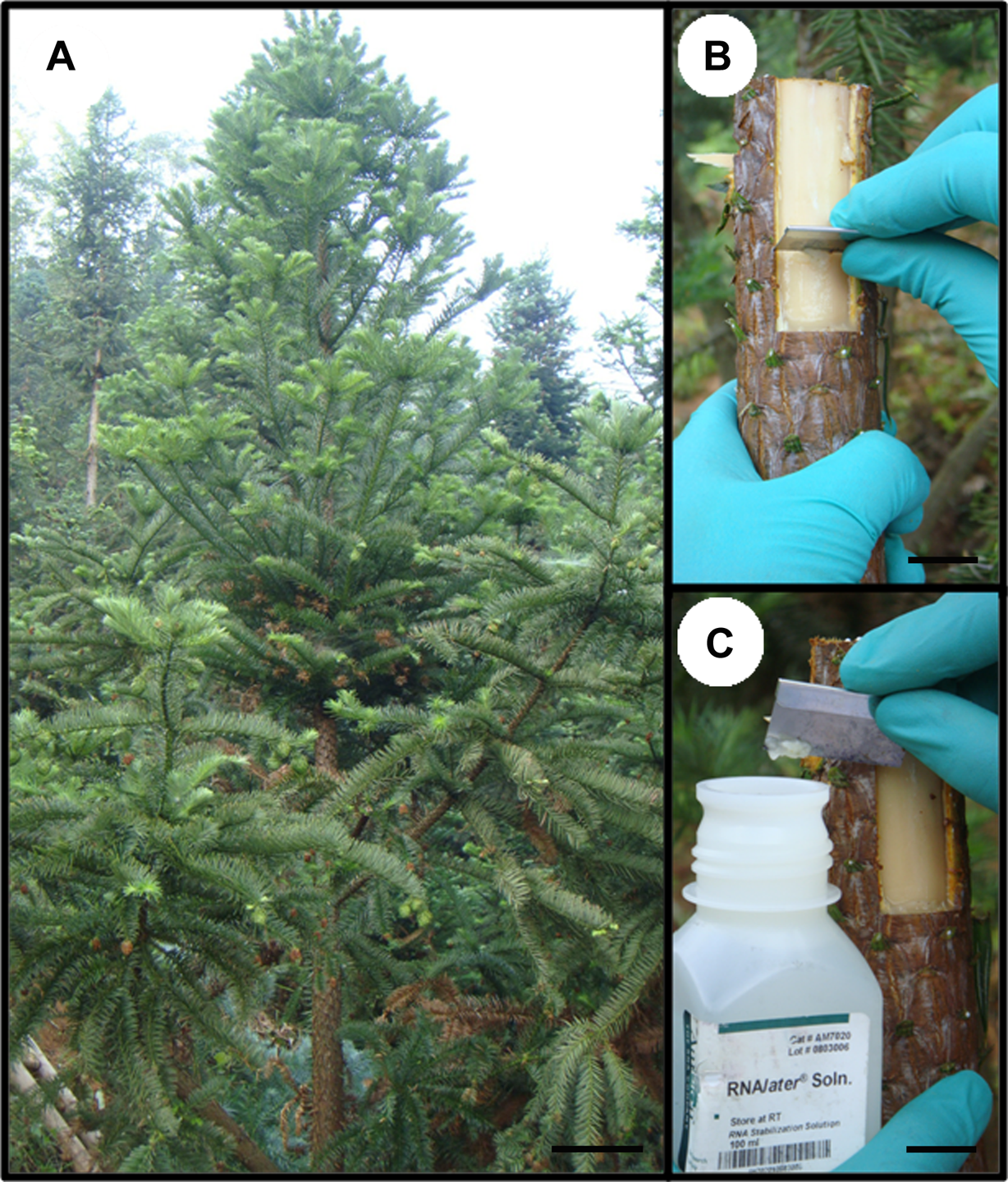

Supplement: Figure S9 — Sampling of Chinese fir cambial tissue. (A) A 7-year-old clone, No. 6421, of Chinese fir. (B) Scraping off the cambial tissues. (C) Collecting and preserving cambial tissues. Bars = 50 cm (A) and 2 cm (B and C). (TIF) [file pone.0071562.s009.tif]
