# Supplementary material for: Transcriptome Characteristics and Six Alternative Expressed Genes Positively Correlated with the Phase Transition of Annual Cambial Activities in Chinese Fir (Cunninghamia lanceolata (Lamb.) Hook)
Source: PLoS One. 2013 Aug 12;8(8):e71562. doi: 10.1371/journal.pone.0071562 (PMC3741379; doi:10.1371/journal.pone.0071562)
Supplement: Table S2 — COG functional annotation mapping of assembled unigenes of the Chinese fir transcriptome. (DOC) [file pone.0071562.s011.doc]

## Table S2. COG functional annotation mapping of assembled unigenes of the Chinese fir transcriptome.

| Code | Functional categories | Number of genes |
| --- | --- | --- |
| A | RNA processing and modification | 131 |
| B | Chromatin structure and dynamics | 217 |
| C | Energy production and conversion | 551 |
| D | Cell cycle control, cell division, chromosome partitioning | 492 |
| E | Amino acid transport and metabolism | 760 |
| F | Nucleotide transport and metabolism | 181 |
| G | Carbohydrate transport and metabolism | 878 |
| H | Coenzyme transport and metabolism | 341 |
| I | Lipid transport and metabolism | 416 |
| J | Translation, ribosomal structure and biogenesis | 871 |
| K | Transcription | 1308 |
| L | Replication, recombination and repair | 1495 |
| M | Cell wall/membrane/envelope biogenesis | 493 |
| N | Cell motility | 59 |
| O | Posttranslational modification, protein turnover, chaperones | 1238 |
| P | Inorganic ion transport and metabolism | 490 |
| Q | Secondary metabolites biosynthesis, transport and catabolism | 425 |
| R | General function prediction only | 2605 |
| S | Function unknown | 787 |
| T | Signal transduction mechanisms | 1015 |
| U | Intracellular trafficking, secretion, and vesicular transport | 357 |
| V | Defense mechanisms | 275 |
| W | Extracellular structures | 4 |
| Y | Nuclear structure | 5 |
| Z | Cytoskeleton | 268 |
