# Supplementary material for: Transcriptome Characteristics and Six Alternative Expressed Genes Positively Correlated with the Phase Transition of Annual Cambial Activities in Chinese Fir (Cunninghamia lanceolata (Lamb.) Hook)
Source: PLoS One. 2013 Aug 12;8(8):e71562. doi: 10.1371/journal.pone.0071562 (PMC3741379; doi:10.1371/journal.pone.0071562)
Supplement: Table S3 — GO mapping of assembled unigenes of the Chinese fir transcriptome. (DOC) [file pone.0071562.s012.doc]

## Table S3. GO mapping of assembled unigenes of the Chinese fir transcriptome.

| Ontology | Function | Number | Percentage |
| --- | --- | --- | --- |
| Biological process | anatomical structure formation | 57 | 0.009262 |
| Biological process | biological adhesion | 6 | 0.000975 |
| Biological process | biological regulation | 428 | 0.069548 |
| Biological process | cell killing | 0 | 0 |
| Biological process | cellular component biogenesis | 82 | 0.013325 |
| Biological process | cellular component organization | 144 | 0.023399 |
| Biological process | cellular process | 2147 | 0.348879 |
| Biological process | death | 11 | 0.001787 |
| Biological process | developmental process | 64 | 0.0104 |
| Biological process | establishment of localization | 482 | 0.078323 |
| Biological process | growth | 12 | 0.00195 |
| Biological process | immune system process | 11 | 0.001787 |
| Biological process | localization | 483 | 0.078486 |
| Biological process | locomotion | 0 | 0 |
| Biological process | metabolic process | 2220 | 0.360741 |
| Biological process | multi-organism process | 49 | 0.007962 |
| Biological process | multicellular organismal process | 80 | 0.013 |
| Biological process | pigmentation | 393 | 0.063861 |
| Biological process | reproduction | 31 | 0.005037 |
| Biological process | reproductive process | 28 | 0.00455 |
| Biological process | response to stimulus | 323 | 0.052486 |
| Biological process | rhythmic process | 0 | 0 |
| Biological process | viral reproduction | 0 | 0 |
| Cellular component | cell | 3923 | 0.637472 |
| Cellular component | cell part | 3922 | 0.637309 |
| Cellular component | envelope | 90 | 0.014625 |
| Cellular component | extracellular region | 86 | 0.013975 |
| Cellular component | extracellular region part | 2 | 0.000325 |
| Cellular component | macromolecular complex | 438 | 0.071173 |
| Cellular component | membrane-enclosed lumen | 64 | 0.0104 |
| Cellular component | organelle | 2902 | 0.471563 |
| Cellular component | organelle part | 390 | 0.063373 |
| Cellular component | symplast | 0 | 0 |
| Cellular component | synapse | 0 | 0 |
| Cellular component | synapse part | 0 | 0 |
| Cellular component | virion | 1 | 0.000162 |
| Cellular component | virion part | 1 | 0.000162 |
| Molecular function | antioxidant | 45 | 0.007312 |
| Molecular function | auxiliary transport protein | 0 | 0 |
| Molecular function | binding | 2659 | 0.432077 |
| Molecular function | catalytic | 2457 | 0.399253 |
| Molecular function | chemoattractant | 0 | 0 |
| Molecular function | chemorepellent | 0 | 0 |
| Molecular function | electron carrier | 97 | 0.015762 |
| Molecular function | enzyme regulator | 16 | 0.0026 |
| Molecular function | metallochaperone | 1 | 0.000162 |
| Molecular function | molecular transducer | 82 | 0.013325 |
| Molecular function | nutrient reservoir | 5 | 0.000812 |
| Molecular function | proteasome regulator | 0 | 0 |
| Molecular function | protein tag | 0 | 0 |
| Molecular function | structural molecule | 88 | 0.0143 |
| Molecular function | transcription regulator | 129 | 0.020962 |
| Molecular function | translation regulator | 28 | 0.00455 |
| Molecular function | transporter | 314 | 0.051024 |
