# Supplementary material for: Transcriptome Characteristics and Six Alternative Expressed Genes Positively Correlated with the Phase Transition of Annual Cambial Activities in Chinese Fir (Cunninghamia lanceolata (Lamb.) Hook)
Source: PLoS One. 2013 Aug 12;8(8):e71562. doi: 10.1371/journal.pone.0071562 (PMC3741379; doi:10.1371/journal.pone.0071562)
Supplement: Table S4 — Mapping of assembled unigenes of the Chinese fir transcriptome to KEGG pathways. (DOC) [file pone.0071562.s013.doc]

## Table S4. Mapping of assembled unigenes of the Chinese fir transcriptome to KEGG pathways.

| # | Pathway | Count (14,402) | Pathway ID |
| --- | --- | --- | --- |
| 1 | Metabolic pathways | 3181 | ko01100 |
| 2 | Plant-pathogen interaction | 1197 | ko04626 |
| 3 | Spliceosome | 841 | ko03040 |
| 4 | Biosynthesis of plant hormones | 695 | ko01070 |
| 5 | Biosynthesis of phenylpropanoids | 567 | ko01061 |
| 6 | Starch and sucrose metabolism | 392 | ko00500 |
| 7 | Biosynthesis of terpenoids and steroids | 381 | ko01062 |
| 8 | Biosynthesis of alkaloids derived from shikimate pathway | 347 | ko01063 |
| 9 | Ubiquitin mediated proteolysis | 343 | ko04120 |
| 10 | Purine metabolism | 333 | ko00230 |
| 11 | Phenylpropanoid biosynthesis | 309 | ko00940 |
| 12 | Biosynthesis of alkaloids derived from ornithine, lysine and nicotinic acid | 307 | ko01064 |
| 13 | Endocytosis | 276 | ko04144 |
| 14 | Pyrimidine metabolism | 272 | ko00240 |
| 15 | Biosynthesis of alkaloids derived from terpenoid and polyketide | 259 | ko01066 |
| 16 | Biosynthesis of alkaloids derived from histidine and purine | 255 | ko01065 |
| 17 | Glycolysis / Gluconeogenesis | 237 | ko00010 |
| 18 | Flavonoid biosynthesis | 213 | ko00941 |
| 19 | Ribosome | 210 | ko03010 |
| 20 | RNA degradation | 206 | ko03018 |
| 21 | Peroxisome | 203 | ko04146 |
| 22 | Cysteine and methionine metabolism | 184 | ko00270 |
| 23 | Oxidative phosphorylation | 180 | ko00190 |
| 24 | Nucleotide excision repair | 180 | ko03420 |
| 25 | ABC transporters | 177 | ko02010 |
| 26 | Amino sugar and nucleotide sugar metabolism | 170 | ko00520 |
| 27 | Limonene and pinene degradation | 158 | ko00903 |
| 28 | Pyruvate metabolism | 157 | ko00620 |
| 29 | Galactose metabolism | 156 | ko00052 |
| 30 | Glycerophospholipid metabolism | 147 | ko00564 |
| 31 | α-Linolenic acid metabolism | 145 | ko00592 |
| 32 | Aminoacyl-tRNA biosynthesis | 142 | ko00970 |
| 33 | DNA replication | 140 | ko03030 |
| 34 | Stilbenoid, diarylheptanoid and gingerol biosynthesis | 138 | ko00945 |
| 35 | Circadian rhythm - plant | 137 | ko04712 |
| 36 | Basal transcription factors | 136 | ko03022 |
| 37 | Fatty acid metabolism | 135 | ko00071 |
| 38 | Glycerolipid metabolism | 131 | ko00561 |
| 39 | Cyanoamino acid metabolism | 131 | ko00460 |
| 40 | Arginine and proline metabolism | 130 | ko00330 |
| 41 | Photosynthesis | 129 | ko00195 |
| 42 | Phosphatidylinositol signaling system | 123 | ko04070 |
| 43 | Carbon fixation in photosynthetic organisms | 119 | ko00710 |
| 44 | Glutathione metabolism | 118 | ko00480 |
| 45 | Nitrogen metabolism | 116 | ko00910 |
| 46 | RNA polymerase | 116 | ko03020 |
| 47 | Base excision repair | 116 | ko03410 |
| 48 | Homologous recombination | 115 | ko03440 |
| 49 | Pentose and glucuronate interconversions | 115 | ko00040 |
| 50 | Inositol phosphate metabolism | 114 | ko00562 |
| 51 | Propanoate metabolism | 113 | ko00640 |
| 52 | Fructose and mannose metabolism | 113 | ko00051 |
| 53 | Mismatch repair | 109 | ko03430 |
| 54 | Tryptophan metabolism | 108 | ko00380 |
| 55 | Alanine, aspartate and glutamate metabolism | 106 | ko00250 |
| 56 | Glycine, serine and threonine metabolism | 106 | ko00260 |
| 57 | Phenylalanine metabolism | 105 | ko00360 |
| 58 | Butanoate metabolism | 101 | ko00650 |
| 59 | Tyrosine metabolism | 100 | ko00350 |
| 60 | Ascorbate and aldarate metabolism | 97 | ko00053 |
| 61 | Valine, leucine and isoleucine degradation | 94 | ko00280 |
| 62 | Zeatin biosynthesis | 93 | ko00908 |
| 63 | Methane metabolism | 90 | ko00680 |
| 64 | Lysine degradation | 88 | ko00310 |
| 65 | N-Glycan biosynthesis | 87 | ko00510 |
| 66 | Linoleic acid metabolism | 86 | ko00591 |
| 67 | Carotenoid biosynthesis | 86 | ko00906 |
| 68 | Citrate cycle (TCA cycle) | 86 | ko00020 |
| 69 | Proteasome | 84 | ko03050 |
| 70 | Ubiquinone and other terpenoid-quinone biosynthesis | 83 | ko00130 |
| 71 | Pentose phosphate pathway | 82 | ko00030 |
| 72 | Biosynthesis of unsaturated fatty acids | 82 | ko01040 |
| 73 | Selenoamino acid metabolism | 80 | ko00450 |
| 74 | Other glycan degradation | 79 | ko00511 |
| 75 | Phenylalanine, tyrosine and tryptophan biosynthesis | 79 | ko00400 |
| 76 | Metabolism of xenobiotics by cytochrome P450 | 77 | ko00980 |
| 77 | Valine, leucine and isoleucine biosynthesis | 74 | ko00290 |
| 78 | Porphyrin and chlorophyll metabolism | 73 | ko00860 |
| 79 | Sphingolipid metabolism | 72 | ko00600 |
| 80 | Terpenoid backbone biosynthesis | 68 | ko00900 |
| 81 | Protein export | 67 | ko03060 |
| 82 | β-Alanine metabolism | 67 | ko00410 |
| 83 | SNARE interactions in vesicular transport | 63 | ko04130 |
| 84 | Fatty acid biosynthesis | 60 | ko00061 |
| 85 | Flavone and flavonol biosynthesis | 60 | ko00944 |
| 86 | Non-homologous end-joining | 57 | ko03450 |
| 87 | Regulation of autophagy | 57 | ko04140 |
| 88 | Histidine metabolism | 56 | ko00340 |
| 89 | Ether lipid metabolism | 55 | ko00565 |
| 90 | Photosynthesis - antenna proteins | 55 | ko00196 |
| 91 | Diterpenoid biosynthesis | 50 | ko00904 |
| 92 | Glyoxylate and dicarboxylate metabolism | 47 | ko00630 |
| 93 | Sulfur metabolism | 44 | ko00920 |
| 94 | Steroid biosynthesis | 43 | ko00100 |
| 95 | Tropane, piperidine and pyridine alkaloid biosynthesis | 40 | ko00960 |
| 96 | Natural killer cell mediated cytotoxicity | 38 | ko04650 |
| 97 | Pantothenate and CoA biosynthesis | 37 | ko00770 |
| 98 | Arachidonic acid metabolism | 37 | ko00590 |
| 99 | Nicotinate and nicotinamide metabolism | 35 | ko00760 |
| 100 | One carbon pool by folate | 32 | ko00670 |
| 101 | Glycosylphosphatidylinositol(GPI)-anchor biosynthesis | 29 | ko00563 |
| 102 | Isoquinoline alkaloid biosynthesis | 27 | ko00950 |
| 103 | Brassinosteroid biosynthesis | 26 | ko00905 |
| 104 | Lysine biosynthesis | 26 | ko00300 |
| 105 | Glycosaminoglycan degradation | 25 | ko00531 |
| 106 | Glucosinolate biosynthesis | 23 | ko00966 |
| 107 | Glycosphingolipid biosynthesis - globo series | 23 | ko00603 |
| 108 | Riboflavin metabolism | 22 | ko00740 |
| 109 | Thiamine metabolism | 21 | ko00730 |
| 110 | Indole alkaloid biosynthesis | 19 | ko00901 |
| 111 | Glycosphingolipid biosynthesis - ganglio series | 17 | ko00604 |
| 112 | Taurine and hypotaurine metabolism | 16 | ko00430 |
| 113 | Folate biosynthesis | 16 | ko00790 |
| 114 | Caffeine metabolism | 16 | ko00232 |
| 115 | Benzoxazinoid biosynthesis | 12 | ko00402 |
| 116 | C5-Branched dibasic acid metabolism | 12 | ko00660 |
| 117 | Polyketide sugar unit biosynthesis | 11 | ko00523 |
| 118 | Biotin metabolism | 11 | ko00780 |
| 119 | Lipoic acid metabolism | 10 | ko00785 |
| 120 | Synthesis and degradation of ketone bodies | 10 | ko00072 |
| 121 | Betalain biosynthesis | 9 | ko00965 |
| 122 | Vitamin B6 metabolism | 8 | ko00750 |
| 123 | Anthocyanin biosynthesis | 7 | ko00942 |
| 124 | Monoterpenoid biosynthesis | 4 | ko00902 |
| 125 | Fatty acid elongation in mitochondria | 2 | ko00062 |
