# Supplementary material for: Transcriptome Characteristics and Six Alternative Expressed Genes Positively Correlated with the Phase Transition of Annual Cambial Activities in Chinese Fir (Cunninghamia lanceolata (Lamb.) Hook)
Source: PLoS One. 2013 Aug 12;8(8):e71562. doi: 10.1371/journal.pone.0071562 (PMC3741379; doi:10.1371/journal.pone.0071562)
Supplement: Table S5 — Candidate genes used to select housekeeping genes. (DOC) [file pone.0071562.s014.doc]

## Table S5. Candidate genes used to select housekeeping genes.

| Gene | Unigene ID | Primer sequence (5'→3') | Putative annotation | ID | *E* value |
| --- | --- | --- | --- | --- | --- |
| Actin | Unigene 33926 | For CTACGCGAAACAGGGTTGTA  Rev CACATACGCATCCTTTGCTT | β-actin | gi|3107919| | 8.00E-28 |
| EF-1α | Unigene 15744 | For GCACTGTTATTGATGCTCCTG  Rev CCAGTGGTGGAGTCAATGAT | elongation factor-1 α | gi|3869088| | 1.00E-101 |
| eIF-3 | Unigene 60891 | For TCTGCATCCCAATGATTTGT  Rev TCTTCAGATGTTGCTTGCCT | eukaryotic initiation factor 3 | gi|18406875| | 9.00E-86 |
| eIF-4A | Unigene 9483 | For AAATACTTGTGTTGGAGGAACAAG  Rev CATATCATAAACCCTACCAGGTGT | eukaryotic initiation factor 4A | gi|152003981| | 5.00E-50 |
| GAPDH | Unigene 41195 | For ATATCGAGCTCGTTGCAGTG  Rev CATCCTTGACCTTGACCTCAT | glyceraldehyde-3-phosphate dehydrogenase, cytosolic | gi|3023813| | 1.00E-34 |
| α-TU | Unigene 12970 | For TGTGCACTGGTATGTTGGTG  Rev TCATCACCCTCAGCAGACTC | α-tubulin | gi|134035504| | 6.00E-14 |
| β-TU | Unigene 29685 | For CTGCCTGTGATATCCCTCCT  Rev TCTCCGGTATACCAATGCAA | β-tubulin | gi|153799895| | 1.00E-136 |
| 40S | Unigene 30833 | For CGTAGCAAGGCTCCAAAGAT  Rev CTTGGCCTTAACAATTCGCT | 40S ribosomal protein S6 | gi|255581734| | 2.00E-133 |
| UBQ | Unigene 889 | For GTGGAAAGCTCCGATACCAT  Rev AGCTGCTTTCCAGCAAAGAT | ubiquitin | gi|111218904| | 4.00E-96 |
