# Supplementary material for: Transcriptome Characteristics and Six Alternative Expressed Genes Positively Correlated with the Phase Transition of Annual Cambial Activities in Chinese Fir (Cunninghamia lanceolata (Lamb.) Hook)
Source: PLoS One. 2013 Aug 12;8(8):e71562. doi: 10.1371/journal.pone.0071562 (PMC3741379; doi:10.1371/journal.pone.0071562)
Supplement: Table S6 — Ranking of candidate genes based on their stability value using geNorm and NormFinder, and their Pearson’s correlation coefficient (r) using BestKeeper. (DOC) [file pone.0071562.s015.doc]

## Table S6. Ranking of candidate genes based on their stability value using geNorm and NormFinder, and their Pearson’s correlation coefficient (r) using BestKeeper.

| Gene | Stability value (geNorm) | Rank | Stability value (NormFinder) | Rank | Pearson’s correlation coefficient (r) (BestKeeper) | Rank |
| --- | --- | --- | --- | --- | --- | --- |
| Actin | 0.037 | 6 | 0.012 | 1 | 0.973 | 9 |
| EF-1α | 0.022 | 3 | 0.036 | 5 | 0.772 | 2 |
| eIF-3 | 0.015 | 1 | 0.027 | 4 | 0.783 | 3 |
| eIF-4A | 0.033 | 5 | 0.015 | 2 | 0.958 | 6 |
| GAPDH | 0.027 | 4 | 0.047 | 6 | 0.677 | 1 |
| α-TU | 0.092 | 9 | 0.097 | 9 | 0.787 | 4 |
| β-TU | 0.075 | 8 | 0.085 | 8 | 0.966 | 8 |
| 40S | 0.052 | 7 | 0.054 | 7 | 0.862 | 5 |
| UBQ | 0.015 | 1 | 0.017 | 3 | 0.965 | 7 |
