# Supplementary material for: Transcriptome Characteristics and Six Alternative Expressed Genes Positively Correlated with the Phase Transition of Annual Cambial Activities in Chinese Fir (Cunninghamia lanceolata (Lamb.) Hook)
Source: PLoS One. 2013 Aug 12;8(8):e71562. doi: 10.1371/journal.pone.0071562 (PMC3741379; doi:10.1371/journal.pone.0071562)
Supplement: Table S7 — The qRT-PCR primers for the 17 homologous genes. (DOC) [file pone.0071562.s016.doc]

## Table S7. The qRT-PCR primers for the 17 candidate genes.

| Gene | Unigene ID | Primer sequence (5'→3') |
| --- | --- | --- |
| ClWOX1 | Unigene 56359 | For TGATAACAGCCCAATCCTCA  Rev GCAGTAGGAGTCCTCATCCC |
| ClWOX4 | Unigene 30112 | For GAATCCAACACCAGAGCAGA  Rev TCTTGCCTTCAATCTTTCCA |
| ClWOX8 | Unigene 58521 | For ATGTGAAAGAGGGAGATGGG  Rev AGATGCTGACAAATGGTTGC |
| ClWOX9 | Unigene 3729 | For CCATTCCGGAGCAAATAGTAA  Rev TTCTGTTTGGGAACCAGTAGAA |
| ClCLV1 | Unigene 20219 | For GTTCTCGACGCCTACAACAA  Rev TGGAGCCACTGAAATAGCTG |
| ClCLV1-like | Unigene 26065 | For TATGAGCCACAGGTCCTTCA  Rev GAAGACATGGAATGAAGATGTGA |
| ClCLV2 | Unigene 60215 | For CAATTCTCTCACTGGCGGTA  Rev AGAGTCGGCGGTAGAGCTAA |
| ClCLV-like | Unigene 57886 | For ATAGCTTTGGCATTGTGCTG  Rev TATCCGGGTGTCCAGAATTT |
| ClCLE12 | Unigene 59778 | For TCCAAGGTATGGTGTGGAGA  Rev ATTCCAATTCTGTGCAGCCT |
| ClREV | Unigene 22939 | For TTGCAAATGAGGCAGGTCTA  Rev AAACCCTGCTGCATCACTTT |
| ClPHB1 | Unigene 56464 | For CCGATCAATTGCTCACAGAG  Rev GGAGAACTCGGCTCCATAAG |
| ClATHB15 | Unigene 38884 | For TTTAGAGCGCCTGTATCGTG  Rev CTCCCTACACCTGCGATTCT |
| ClSHR | Unigene 60570 | For GGACGAAGCCCTAGCAATTA  Rev AGGTCCATTTCGTCTTCCAC |
| ClSCR | Unigene 48261 | For TGAGCTATGCTGGCTCATTC  Rev ACATGGCGCTCAATACTGTC |
| ClPIN1-like | Unigene 38615 | For GGAAATTCCGTTGCAACTTT  Rev TATGGCTACACGGAGAAGCA |
| ClAUX | Unigene 13481 | For CCTTTCTTTGGCCCTATCAA  Rev CTGGCAAGAGCTGTCCTGTA |
| ClARR7 | Unigene 60762 | For TCTTAAGGCGGATCTGTGTG  Rev GCTGGCCTCTCTCTGATTCT |
