# Supplementary material for: Transcriptome Characteristics and Six Alternative Expressed Genes Positively Correlated with the Phase Transition of Annual Cambial Activities in Chinese Fir (Cunninghamia lanceolata (Lamb.) Hook)
Source: PLoS One. 2013 Aug 12;8(8):e71562. doi: 10.1371/journal.pone.0071562 (PMC3741379; doi:10.1371/journal.pone.0071562)
Supplement: Table S8 — Primers for cloning ClWOX1 and ClWOX4 . (DOC) [file pone.0071562.s017.doc]

## Table S8. Primers for cloning *ClWOX1* and *ClWOX4*.

| Usage | Primer name | Primer sequence (5'→3') |
| --- | --- | --- |
| 3' RACE | ClWOX1-3R | CTCCTCACAACTCAGGCGCTATGG |
|  | ClWOX4-3R | GAGGACACCCAACGCAGAACAGATAGA |
| 5' RACE | ClWOX1-5R | GCTCCTGTGCTATTCAAATCAGACCC |
|  | ClWOX4-5R | AAGGAGGAAGAAGTGAAGATAGGCTGA |
| Complete sequence | ClWOX1-CS-F | ATGGGAGGAATGGAGAGGTTAACAA |
|  | ClWOX1-CS-R | CTATTCCCAACAACTAAGAGAGAGG |
|  | ClWOX4-CS-F | ATGGAGGCTAGAATGGCAAGCATG |
|  | ClWOX4-CS-R | TCAAATGGAAAATAAAGAGGACT |
