# Supplementary material for: Transcriptome Characteristics and Six Alternative Expressed Genes Positively Correlated with the Phase Transition of Annual Cambial Activities in Chinese Fir (Cunninghamia lanceolata (Lamb.) Hook)
Source: PLoS One. 2013 Aug 12;8(8):e71562. doi: 10.1371/journal.pone.0071562 (PMC3741379; doi:10.1371/journal.pone.0071562)
Supplement: Table S9 — Protein names and accession numbers of 108 WOX. (DOC) [file pone.0071562.s018.doc]

## Table S9. Protein names and accession numbers of 108 WOX.

|  | Accession Number | Protein name | Accession Number | Protein name | Accession Number |
| --- | --- | --- | --- | --- | --- |
| AtWUS | NP_565429.1 | PtWOX13B | XP_002306983.1 | ZmWOX9B | CAM32351.1 |
| AtWOX1 | NP_188428.3 | PtWOX13C | XP_002301917.1 | ZmWOX9C | CAM32352.1 |
| AtWOX2 | NP_200742.2 | SbWUS1 | XP_002448707.1 | ZmWOX11 | ACG26290.1 |
| AtWOX3 | NP_180429.1 | SbWOX2 | XP_002440499.1 | ZmWOX11A | ACR38139.1 |
| AtWOX4 | NP_175145.2 | SbWOX3B | XP_002449019.1 | ZmWOX11B | NP_001147238.1 |
| AtWOX5 | NP_187735.2 | SbWOX4 | XP_002448640.1 | ZmWOX13 | ACF83546.1 |
| AtWOX6 | NP_565263.1 | SbWOX5 | XP_002458739.1 | ZmWOX14 | ACG33593.1 |
| AtWOX7 | NP_196196.1 | SbWOX6 | XP_002465335.1 | PhWUS | Q8LL11.1 |
| AtWOX8 | NP_199410.2 | SbWOX8 | XP_002456540.1 | PhWOX2 | ACA64094.1 |
| AtWOX9 | NP_180944.2 | SbWOX9 | XP_002458781.1 | PhWOX3 | ACU68503.1 |
| AtWOX10 | NP_173494.1 | SbWOX10 | XP_002444095.1 | PhWOX4 | ACA64095.1 |
| AtWOX11 | NP_187016.2 | SbWOX11 | XP_002461234.1 | SlWUS | ADZ13564.1 |
| AtWOX12 | NP_197283.2 | SbWOX12 | XP_002440254.1 | SlWOX4 | ACJ61689.1 |
| AtWOX13 | NP_195280.1 | VvWUS | XP_002266323.1 | NjWUS | CAT03215.1 |
| AtWOX14 | NP_173493.2 | VvWOX1 | XP_002280774.2 | NjWOX5 | CAT03216.1 |
| OsWUS | CAM32354.1 | VvWOX2 | XP_002281161.1 | AmROA | Q6YBV1.1 |
| OsWOX1A | Q7XM13.2 | VvWOX3 | XP_002281707.1 | CsWUS | ADP02393.1 |
| OsWOX1B | Q33DK0.2 | VvWOX4 | XP_002284927.1 | MtWUS | ACK77479.1 |
| OsWOX2 | Q5W7C3.1 | VvWOX5 | XP_002276008.2 | PsaWOX5 | AEX88469.1 |
| OsNS | Q33DK1.1 | VvWOX8 | XP_002272420.1 | SrWUS | ABS01330.1 |
| OsWOX4 | Q25AM2.1 | VvWOX9 | XP_002273188.1 | GbWUS | CAT02906.1 |
| OsWOX5 | Q8LR86.1 | VvWOX11 | XP_002269282.2 | GbWOX2 | CAT02902.2 |
| OsWOX6 | Q10M29.1 | VvWOX13 | XP_002272863.1 | GbWOX3A | CAT02903.2 |
| OsWOX7 | Q0JKK6.2 | VvWOX13B | XP_002279942.1 | GbWOX3B | CAT02904.1 |
| OsWOX8 | Q5QMM3.1 | ZmWUS1 | NP_001105960.1 | GgWUS | CAT02932.1 |
| OsWOX9 | Q8W0F1.1 | ZmWUS2 | NP_001105961.1 | GgWOX3 | CAT02931.1 |
| OsWOX10 | Q6Z3L4.1 | ZmWOX2A | CAM32345.1 | PsWUS | CAT02938.1 |
| OsWOX11 | Q0D3I7.1 | ZmWOX3A | CAM32346.1 | PsWOX2 | CAT02937.2 |
| OsWOX12 | A3B6V0.2 | ZmWOX3B | CAM33396.1 | PsWOX3 | CAT02936.1 |
| OsWOX13 | A3BKM2.1 | ZmNS1 | Q70UV1.1 | PaWOX2 | CAL18267.1 |
| PtWUS | CAJ84139.1 | ZmNS2 | Q6S3I3.1 | PaWOX8/9 | ADF42580.1 |
| PtWUSB | XP_002327757.1 | ZmWOX4A | CAM32347.1 | PsitWOX4 | ABR17078.1 |
| PtWUS1 | ACO55493.1 | ZmWOX4B | ACG36393.1 | PsitWOX13 | ABK25160.1 |
| PtWUS2 | ACO55494.1 | ZmWOX5A | CAM32348.1 | PcWOX2 | ADR10436.1 |
| PtWOX4B | XP_002301170.1 | ZmWOX5B | CAM32349.1 | ClWOX1 |  |
| PtWOX9B | XP_002331965.1 | ZmWOX9A | CAM32350.1 | ClWOX4 |  |
